# Supplementary material for: Single-base substitutions switch RNA tertiary allostery between positive and negative coupling
Source: bioRxiv. 2026 Aug 10:2026.08.09.743768. Preprint. [Version 1] doi: 10.64898/2026.08.09.743768 (PMC13640659; doi:10.64898/2026.08.09.743768)
Supplement: Supplementary file 1 [file NIHPP2026.08.09.743768v1-supplement-1.pdf]

bioRxiv preprint doi: https://doi.org/10.64898/2026.08.09.743768; this version posted August 11, 2026. The copyright holder for this preprint (which was not certified by peer review) is the author/funder, who has granted bioRxiv a license to display the preprint in perpetuity. It is made available under a CC-BY 4.0 International license.

Supplemental Results

Supplemental Results 1: Helix-only negative control and classification thresholds.

Helix sequence can modulate tertiary-contact stability (43), so we screened 3,590 variants in which only the helix base pairs were randomized, keeping the kink-turn, TL/TLR, and aptamer intact. This pool serves two roles. As a noise model, it has a tight ΔMF(TL/TLR) distribution (Methods). As a negative control, it tests whether helix-sequence variation alone can switch the contact.

Under the classification cutoffs, no helix variant crossed the Switch-Off threshold, two crossed Always-Off, and 110 crossed Switch-On. These 112 apparent calls share a structural signature of weak global folding: weaker TL-helix folding free energy (means −11.8 and −10.6 kcal/mol vs −13.3 kcal/mol for the Always-On majority), more G·U wobbles (2.3 and 4.0 vs 1.8 pairs), and elevated total ΔMF summed over all helix A and C positions (Mann-Whitney $p < 2 \times 10^{-5}$ for each feature in the Switch-On). The breadth of this total-ΔMF signal, present across every helix rather than localized at the TL/TLR, indicates AMP-induced stabilization of the global secondary structure of these weakly folded variants rather than TL/TLR-specific coupling. Helix-sequence variation alone therefore does not generate ligand-induced TL/TLR allostery in this scaffold.

bioRxiv preprint doi: https://doi.org/10.64898/2026.08.09.743768; this version posted August 11, 2026. The copyright holder for this preprint (which was not certified by peer review) is the author/funder, who has granted bioRxiv a license to display the preprint in perpetuity. It is made available under a CC-BY 4.0 International license.

Supplemental Figures

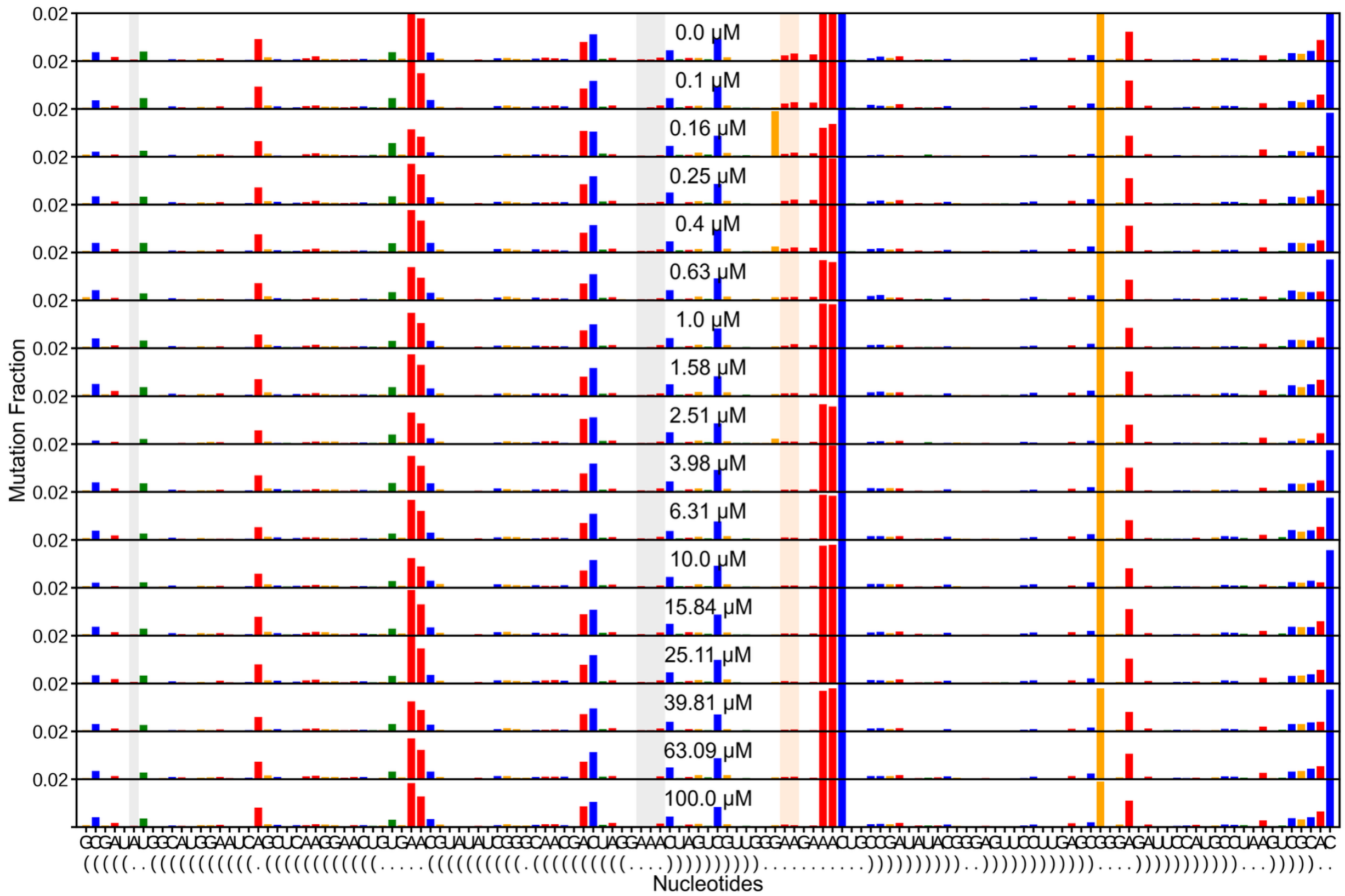

Figure S1: Wild-type per-nucleotide reactivity across the AMP titration, replicate 1

Full per-nucleotide DMS-MaPseq reactivity for the wild-type construct at each point of the AMP titration (replicate 1), stacked from low AMP (top) to high AMP (bottom); each row is the mutation fraction at every nucleotide for one AMP concentration. Gray shading marks the TL/TLR docking reporters (the GAAA tetraloop adenines and the receptor adenine) and orange shading the ATP-aptamer binding-site adenines. Aptamer reactivity falls as AMP rises, reporting ligand binding, while the TL/TLR reporters stay low and roughly constant, so the tertiary contact remains docked across the titration. This is the raw per-replicate data underlying the averaged wild-type curves in Figure 3.

bioRxiv preprint doi: https://doi.org/10.64898/2026.08.09.743768; this version posted August 11, 2026. The copyright holder for this preprint (which was not certified by peer review) is the author/funder, who has granted bioRxiv a license to display the preprint in perpetuity. It is made available under a CC-BY 4.0 International license.

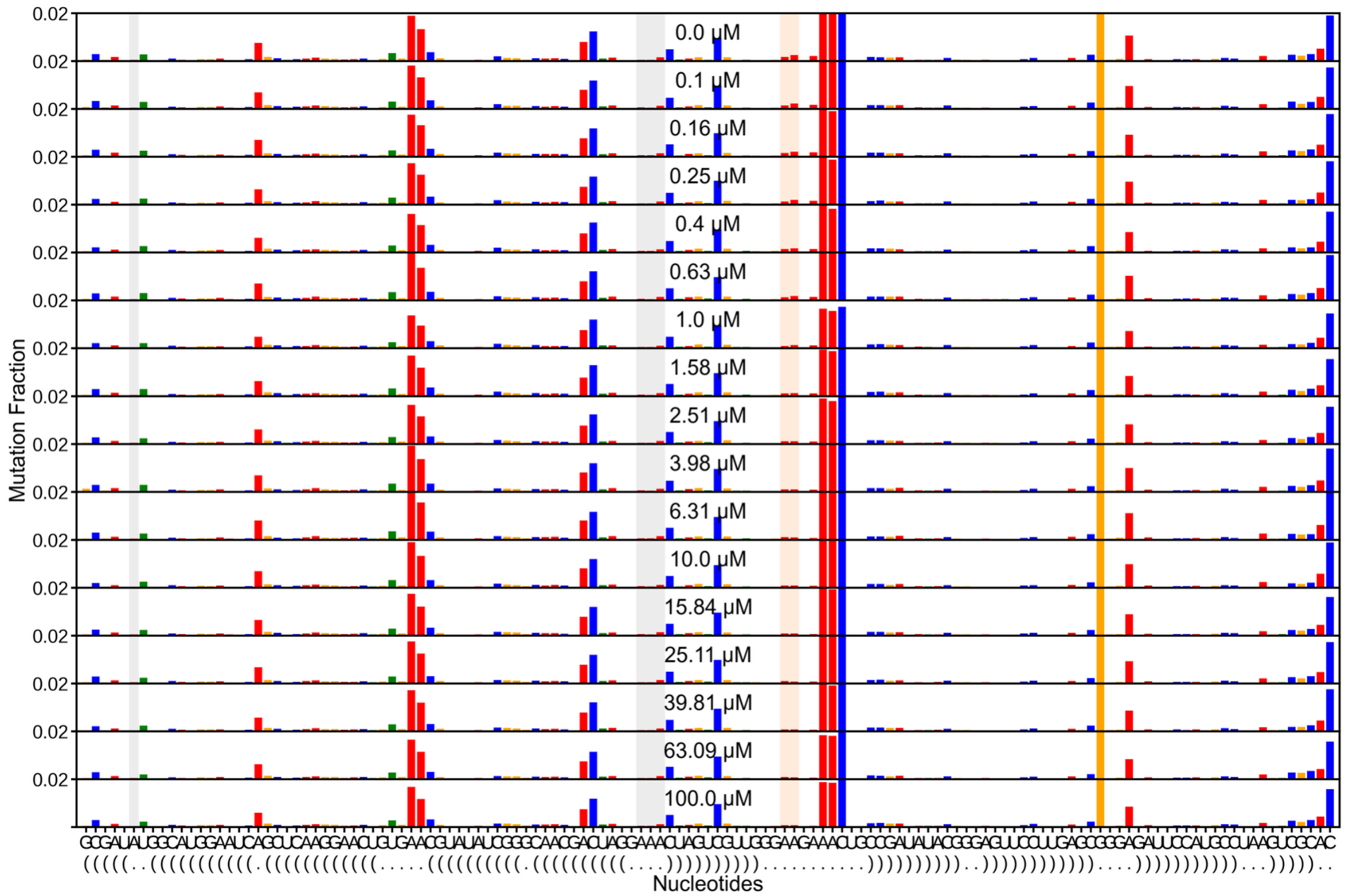

Figure S2: Wild-type per-nucleotide reactivity across the AMP titration, replicate 2

As in Figure S1 for the second wild-type replicate. Full per-nucleotide DMS-MaPseq reactivity at each AMP concentration, with the TL/TLR docking reporters shaded gray and the ATP-aptamer binding-site adenines shaded orange. Aptamer reactivity falls with AMP while TL/TLR reactivity stays low, reproducing the docked, weakly coupled wild-type behavior.

bioRxiv preprint doi: https://doi.org/10.64898/2026.08.09.743768; this version posted August 11, 2026. The copyright holder for this preprint (which was not certified by peer review) is the author/funder, who has granted bioRxiv a license to display the preprint in perpetuity. It is made available under a CC-BY 4.0 International license.

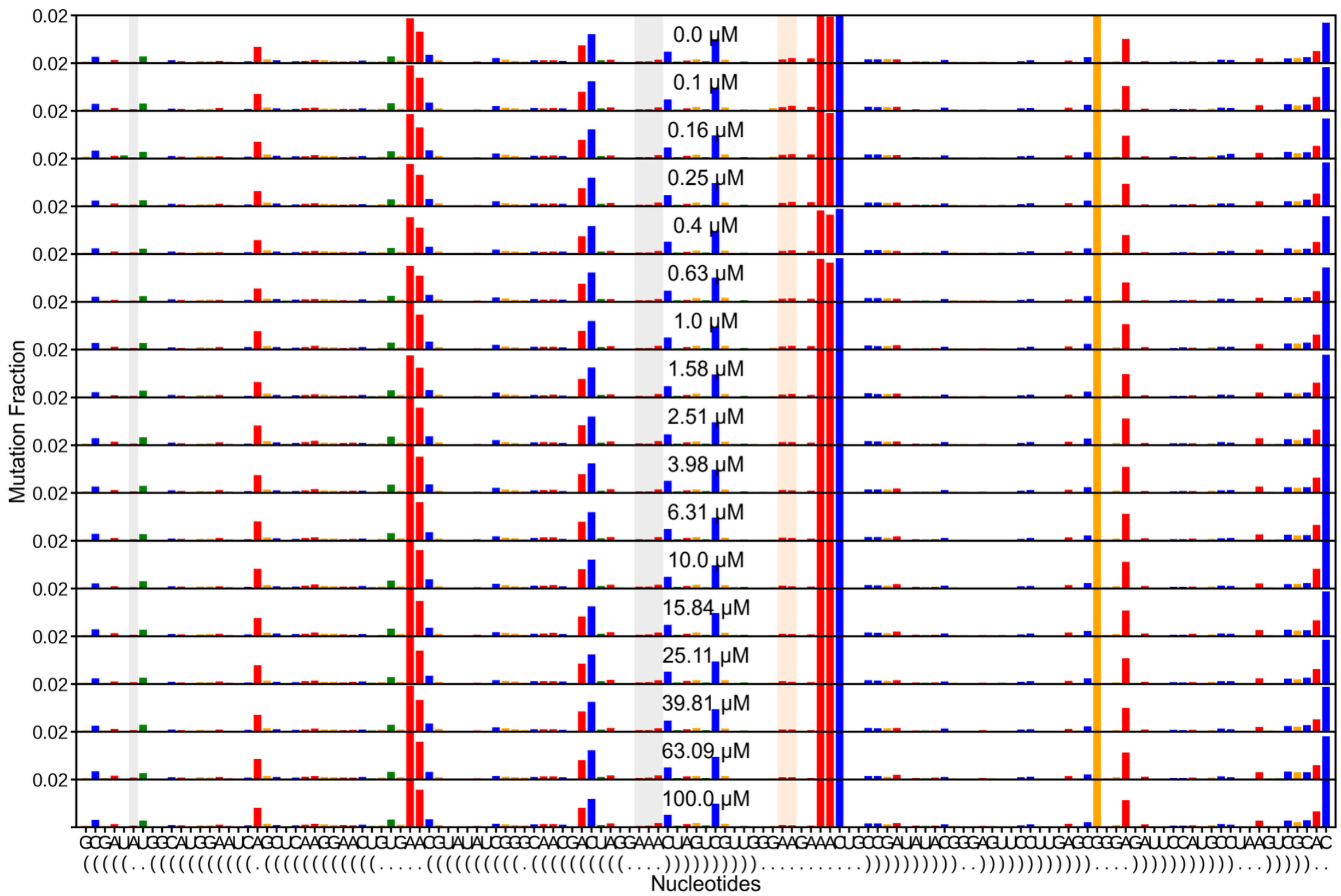

Figure S3: Wild-type per-nucleotide reactivity across the AMP titration, replicate 3

As in Figure S1 for the third wild-type replicate. The aptamer adenines (orange) lose reactivity as AMP increases and the TL/TLR reporters (gray) remain low throughout, consistent across all three replicates.

bioRxiv preprint doi: https://doi.org/10.64898/2026.08.09.743768; this version posted August 11, 2026. The copyright holder for this preprint (which was not certified by peer review) is the author/funder, who has granted bioRxiv a license to display the preprint in perpetuity. It is made available under a CC-BY 4.0 International license.

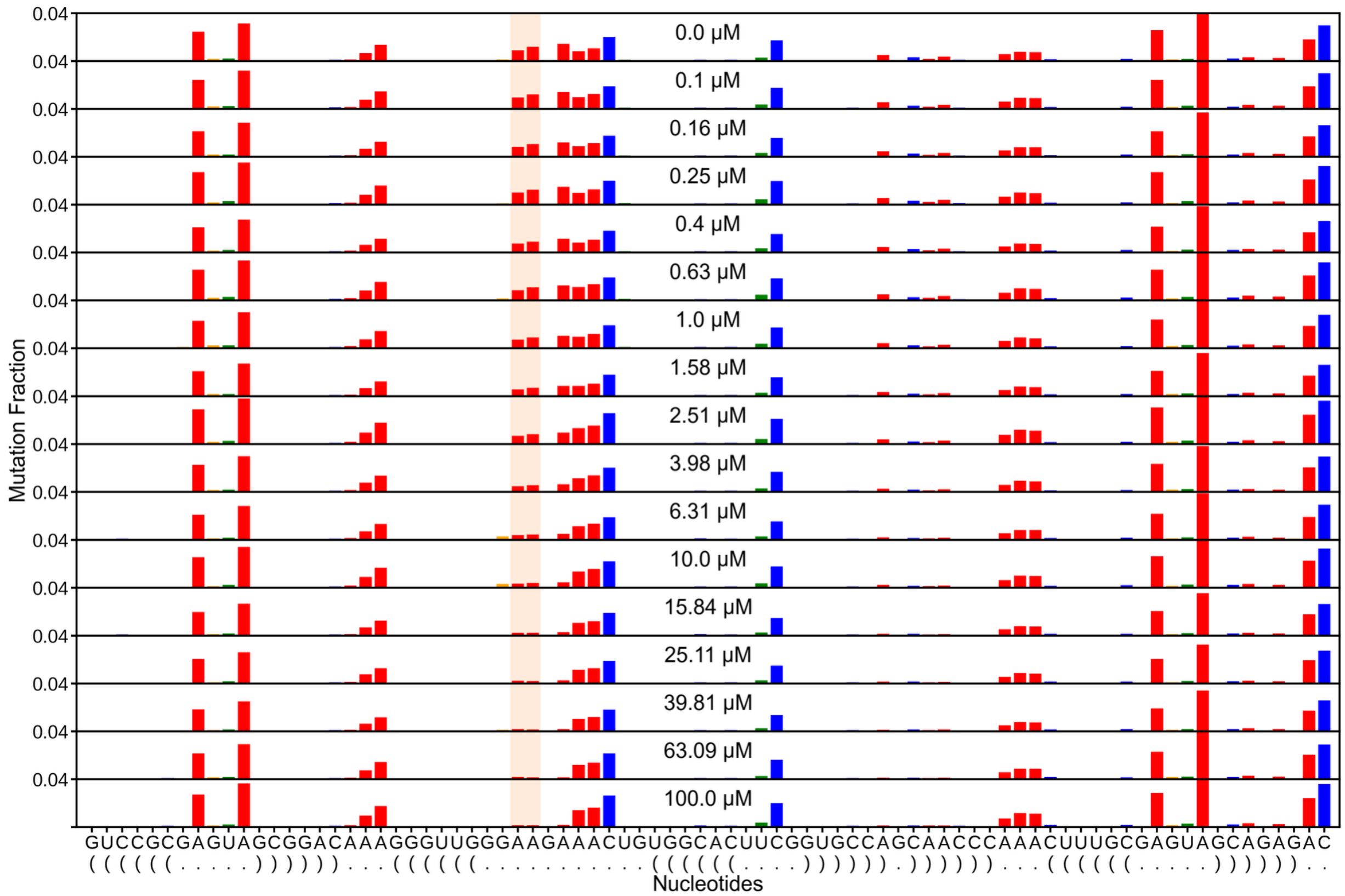

Figure S4: ATP-aptamer-alone per-nucleotide reactivity across the AMP titration, replicate 1

Full per-nucleotide DMS-MaPseq reactivity for the isolated ATP aptamer (no TL/TLR present) at each point of the AMP titration (replicate 1), stacked by AMP concentration. Orange shading marks the binding-site adenines, whose reactivity falls as AMP rises, reporting ligand binding by the aptamer on its own. These per-replicate profiles underlie the aptamer dissociation-constant fit.

bioRxiv preprint doi: https://doi.org/10.64898/2026.08.09.743768; this version posted August 11, 2026. The copyright holder for this preprint (which was not certified by peer review) is the author/funder, who has granted bioRxiv a license to display the preprint in perpetuity. It is made available under a CC-BY 4.0 International license.

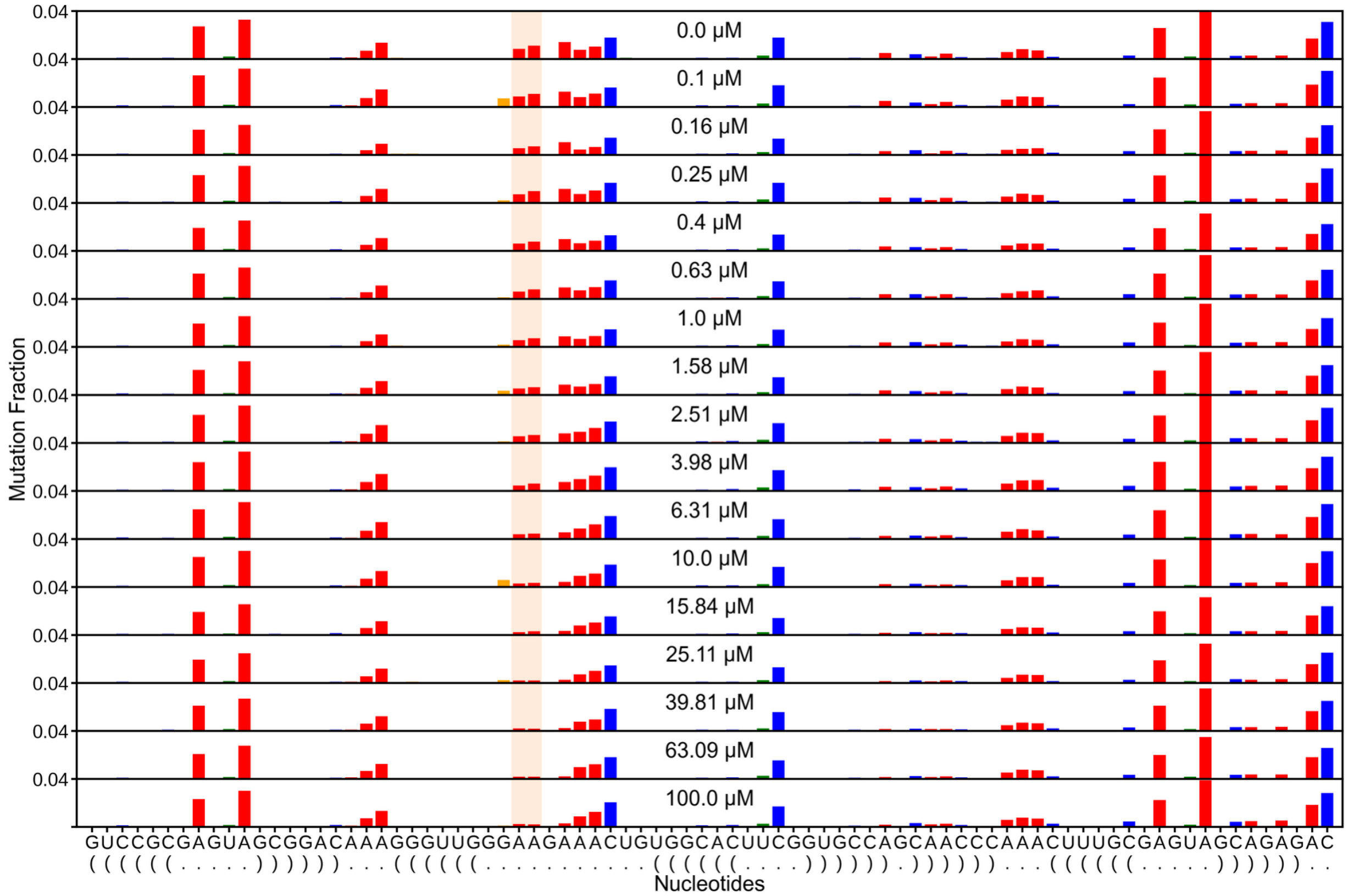

Figure S5: ATP-aptamer-alone per-nucleotide reactivity across the AMP titration, replicate 2

As in Figure S4 for the second ATP-aptamer-alone replicate. The binding-site adenines (orange) are progressively protected as AMP increases, reproducing the isolated-aptamer binding response.

bioRxiv preprint doi: https://doi.org/10.64898/2026.08.09.743768; this version posted August 11, 2026. The copyright holder for this preprint (which was not certified by peer review) is the author/funder, who has granted bioRxiv a license to display the preprint in perpetuity. It is made available under a CC-BY 4.0 International license.

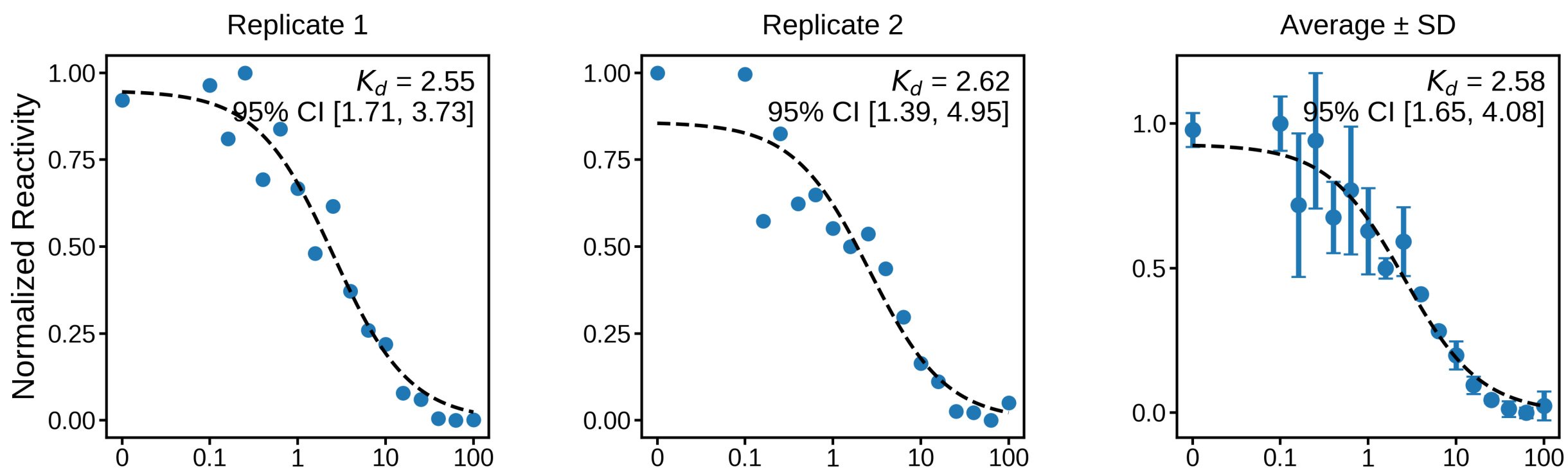

Figure S6: ATP-aptamer-alone AMP binding affinity

Apparent dissociation constant of the isolated ATP aptamer (no TL/TLR). Mean reactivity of the two AMP-sensitive binding-site adenines, normalized to [0, 1], plotted versus AMP concentration for each replicate and for the inter-replicate average (± SD). Dashed lines are three-parameter Hill fits, and the apparent Kd from each fit is annotated. Averaging the replicates gives K_d = 2.58 ± 0.67 μM, consistent across replicates (2.55 ± 0.58 μM and 2.62 ± 0.90 μM).

bioRxiv preprint doi: https://doi.org/10.64898/2026.08.09.743768; this version posted August 11, 2026. The copyright holder for this preprint (which was not certified by peer review) is the author/funder, who has granted bioRxiv a license to display the preprint in perpetuity. It is made available under a CC-BY 4.0 International license.

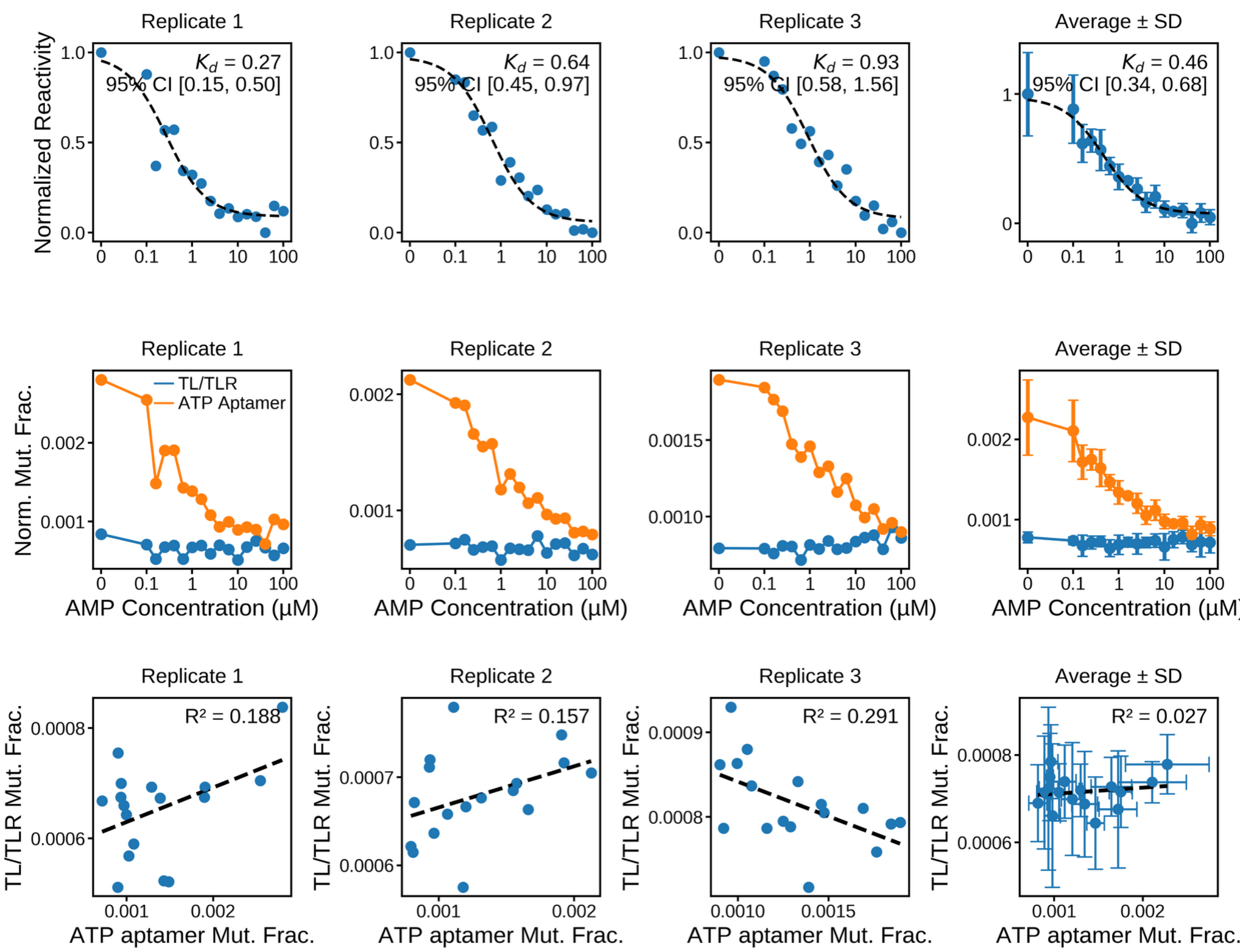

Figure S7: Wild-type AMP binding affinity and weak coupling to TL/TLR docking

(Top) Mean reactivity of the AMP-sensitive ATP-aptamer binding-site adenines, normalized to [0, 1], versus AMP concentration for each wild-type replicate and for the inter-replicate average (± SD). Dashed lines are three-parameter Hill fits, and the apparent Kd from each fit is annotated. The replicate fits give K_d = 0.27 ± 0.10, 0.64 ± 0.14, and 0.93 ± 0.24 μM, and averaging the replicates gives K_d = 0.46 ± 0.09 μM (the value reported in Figure 1C). (Middle) Per-nucleotide normalized mutation fraction across the same titration for the TL/TLR docking reporters (blue) and the ATP-aptamer binding-site adenines (orange). Aptamer reactivity falls as AMP rises, reporting binding, while the TL/TLR reporters stay low and flat, so the tertiary contact stays docked throughout. (Bottom) TL/TLR docking reactivity versus ATP-aptamer reactivity across the titration, per replicate and averaged, with the coefficient of determination annotated.

bioRxiv preprint doi: https://doi.org/10.64898/2026.08.09.743768; this version posted August 11, 2026. The copyright holder for this preprint (which was not certified by peer review) is the author/funder, who has granted bioRxiv a license to display the preprint in perpetuity. It is made available under a CC-BY 4.0 International license.

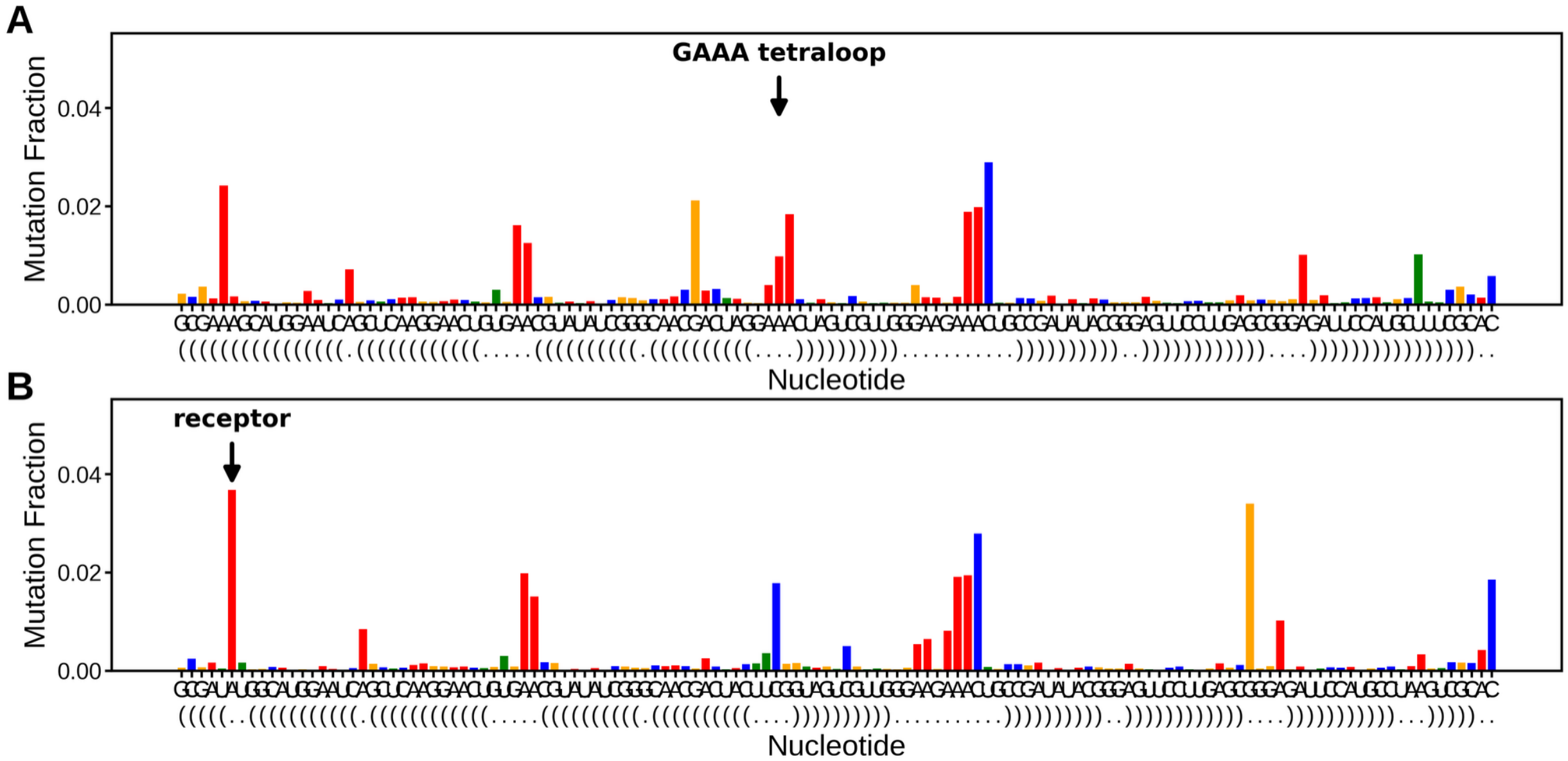

Figure S8: Non-docking controls define the undocked TL/TLR reactivity baseline

Per-nucleotide DMS-MaPseq mutation fraction for control constructs in which the TL/TLR tertiary contact cannot form, so its docking-reporter adenines are fully solvent-exposed. (A) A control probing the GAAA tetraloop (arrow): the tetraloop adenines are highly reactive. (B) A control probing the receptor (arrow): the receptor adenine receptor is highly reactive. These high, undocked reactivities set the reference for a fully open contact and anchor the upper end of the docking scale used to normalize TL/TLR reactivity throughout the study. The dot-bracket secondary structure is drawn beneath each panel.

bioRxiv preprint doi: https://doi.org/10.64898/2026.08.09.743768; this version posted August 11, 2026. The copyright holder for this preprint (which was not certified by peer review) is the author/funder, who has granted bioRxiv a license to display the preprint in perpetuity. It is made available under a CC-BY 4.0 International license.

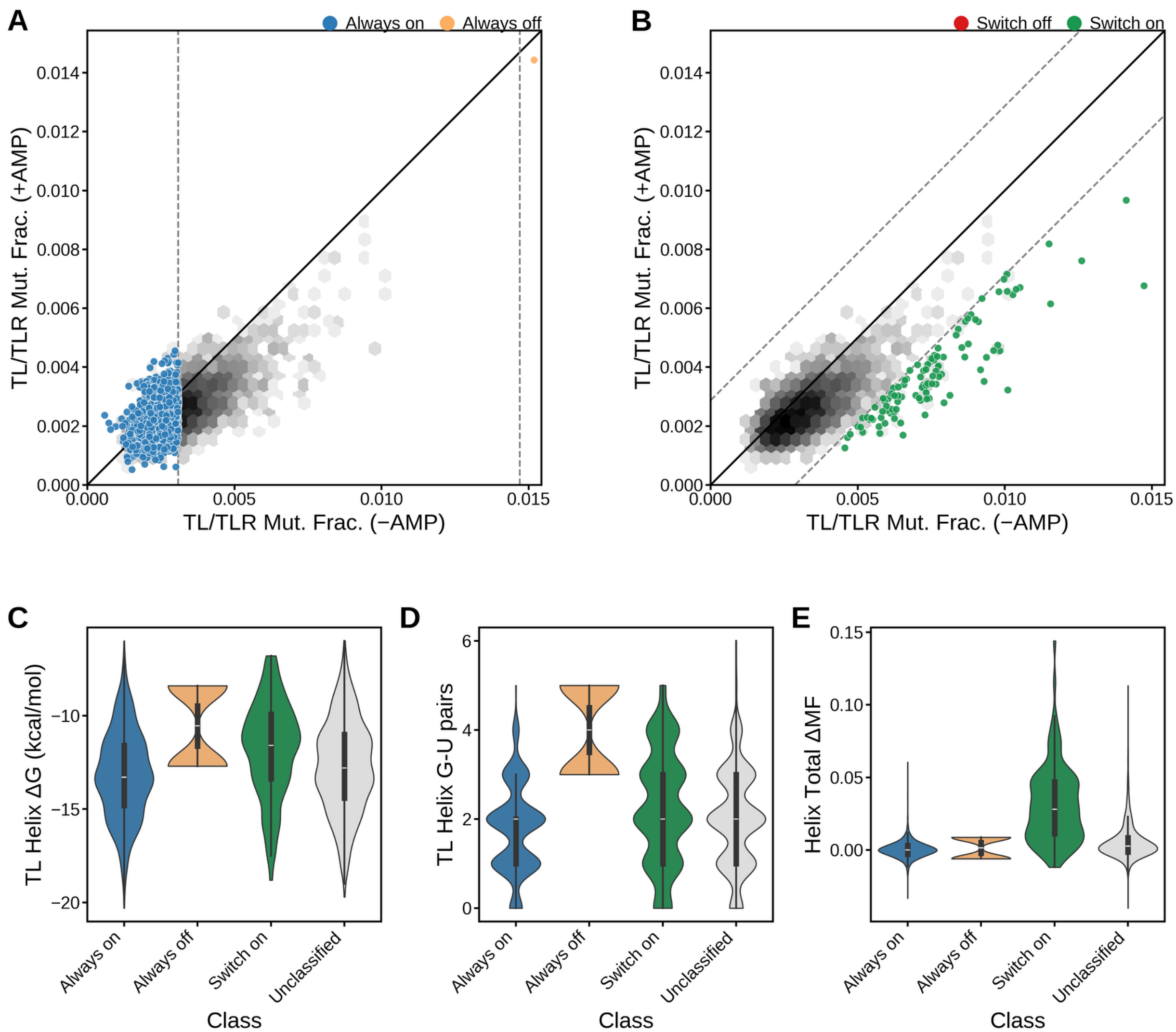

Figure S9: Helix sequence background does not predict switching phenotype

(A and B) TL/TLR reactivity without AMP versus with AMP for all junction variants (gray density), as in Figure 2, with (A) Always-On (blue) and Always-Off (orange) and (B) Switch-Off (red) and Switch-On (green) overlaid. Dashed lines are the same baseline cutoffs (A) and ±3σ helix TL/TLR variation cutoffs (B). (C to E) Distributions of helix-background properties by phenotype class (Always-On blue, Always-Off orange, Switch-On green, Unclassified gray): (C) predicted TL-helix folding free energy ΔG, (D) number of G·U wobble pairs in the TL helix, and (E) total helix ΔMF. The class distributions overlap broadly in ΔG and G·U content, so the randomized helix background does not separate the phenotypes. The only class-linked trend is the elevated helix ΔMF of Switch-On variants in (E). Phenotype is therefore set by junction identity, not by the surrounding helix sequence.

bioRxiv preprint doi: https://doi.org/10.64898/2026.08.09.743768; this version posted August 11, 2026. The copyright holder for this preprint (which was not certified by peer review) is the author/funder, who has granted bioRxiv a license to display the preprint in perpetuity. It is made available under a CC-BY 4.0 International license.

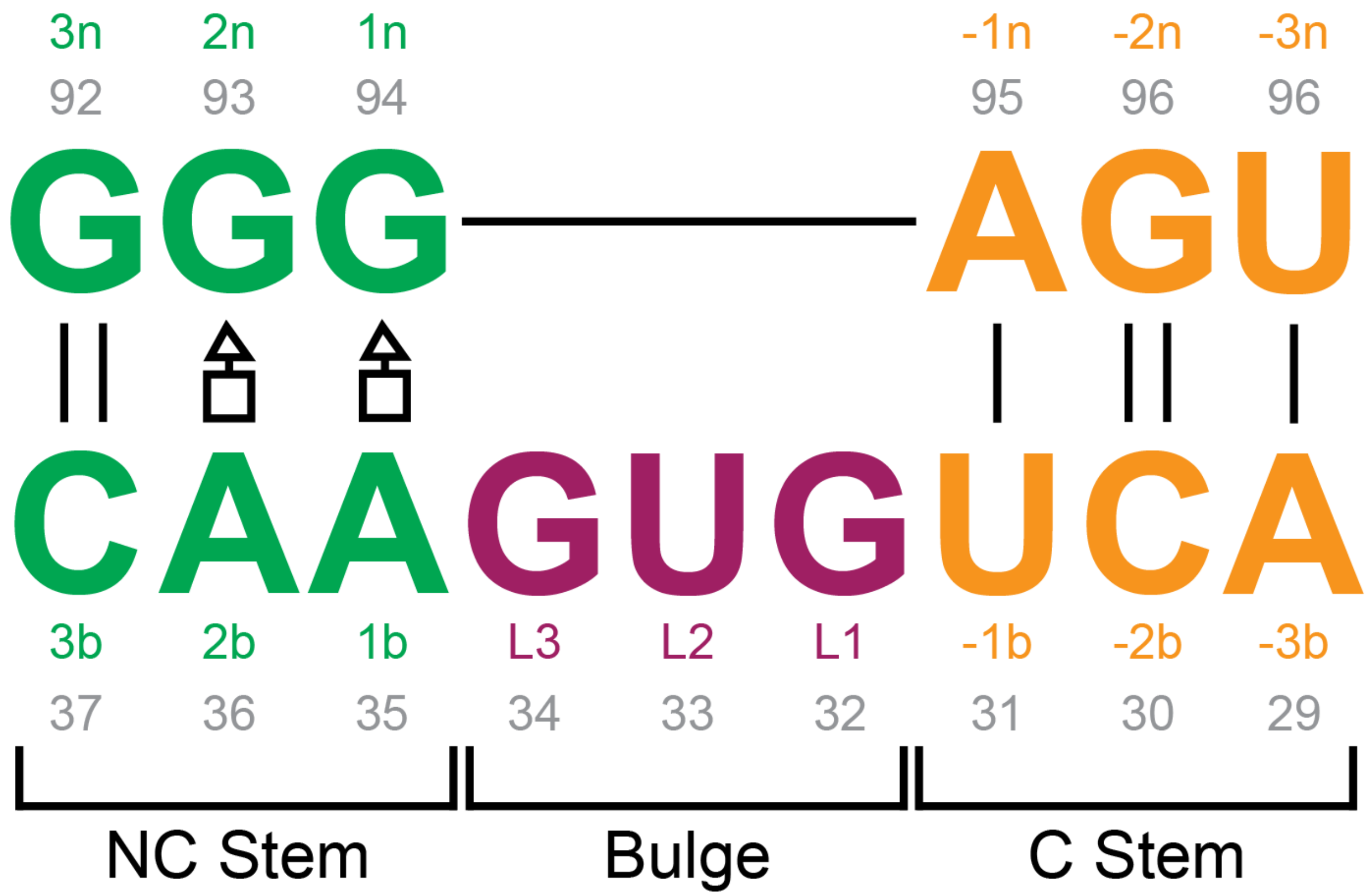

Figure S10: Kink-turn nomenclature and residue numbering

Kink-turn positions in atp_ttr_3, numbered by the standard Leontis-Westhof and Lilley convention. The three-nucleotide bulge is L1 to L3 (positions 32 to 34). Base pairs are counted outward from the bulge: into the NC-stem as 1b·1n (35:94), 2b·2n (36:93), and 3b·3n (37:92), and into the C-stem as -1b:-1n (31:95) and beyond, where b and n denote the bulged and non-bulged strands. The two conserved sheared A·G pairs (1b·1n and 2b·2n) are marked with Leontis-Westhof symbols (open square, Hoogsteen edge; open triangle, sugar edge), and Watson-Crick pairs with vertical lines. Numbers by each base give its atp_ttr_3 residue position.

bioRxiv preprint doi: https://doi.org/10.64898/2026.08.09.743768; this version posted August 11, 2026. The copyright holder for this preprint (which was not certified by peer review) is the author/funder, who has granted bioRxiv a license to display the preprint in perpetuity. It is made available under a CC-BY 4.0 International license.

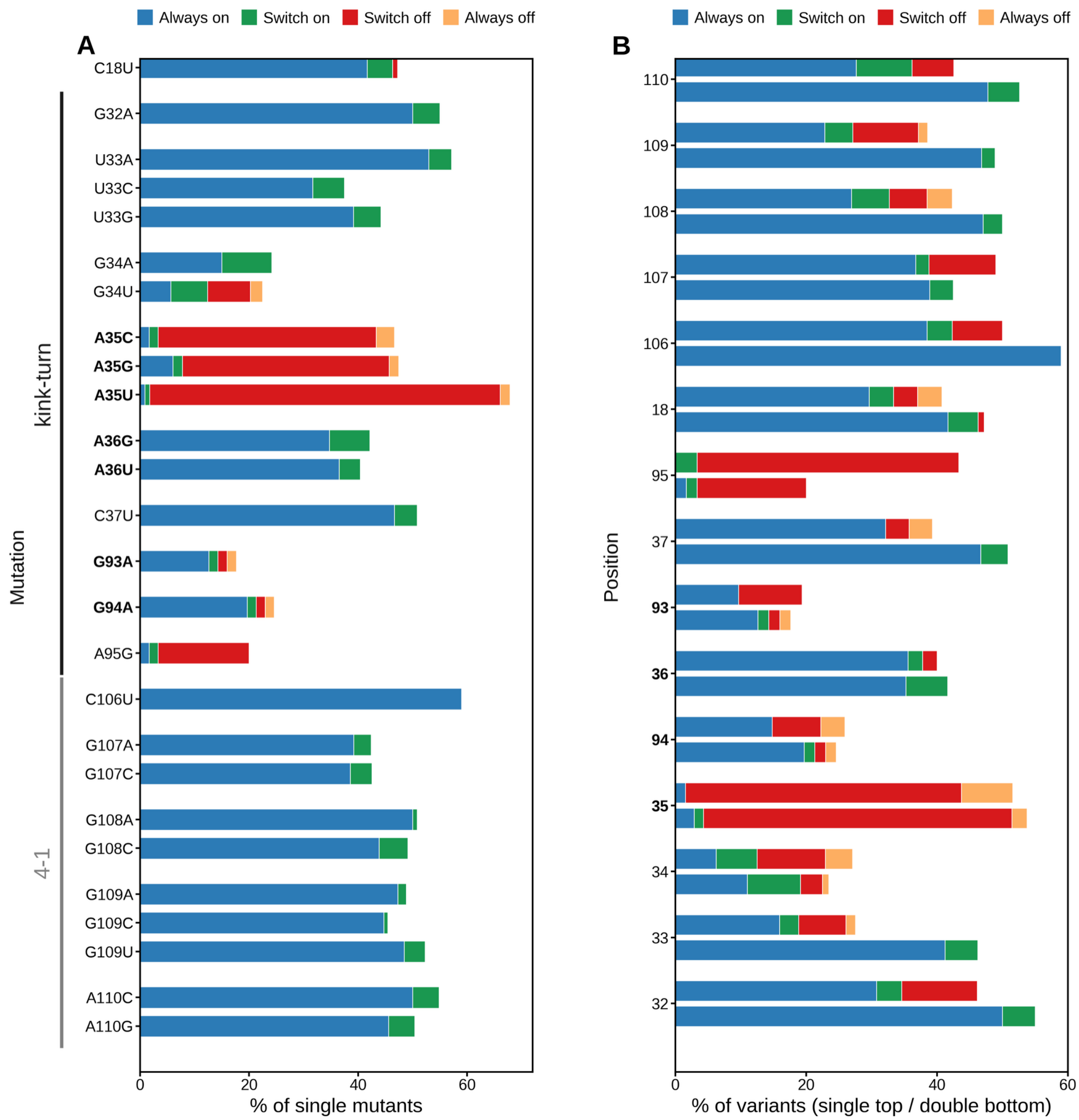

Figure S11: Junction single mutations map to the allosteric regimes by position

(A) Regime composition of each single junction mutation across roughly 120 randomized helix backgrounds, colored by class (Always-On blue, Switch-On green, Switch-Off red, Always-Off orange). Mutations are ordered along the construct and grouped by junction (kink-turn versus 4-1). The kink-turn 1b position dominates: A35C, A35G, and A35U are largely Switch-Off, and A95G at the −1n position is also enriched for Switch-Off, while the 4-1 junction mutations (106 to 110)

bioRxiv preprint doi: https://doi.org/10.64898/2026.08.09.743768; this version posted August 11, 2026. The copyright holder for this preprint (which was not certified by peer review) is the author/funder, who has granted bioRxiv a license to display the preprint in perpetuity. It is made available under a CC-BY 4.0 International license.

are almost uniformly Always-On. (B) The same composition per position, comparing single mutants (top bar) with double mutants (bottom bar) that carry a second junction change.

bioRxiv preprint doi: https://doi.org/10.64898/2026.08.09.743768; this version posted August 11, 2026. The copyright holder for this preprint (which was not certified by peer review) is the author/funder, who has granted bioRxiv a license to display the preprint in perpetuity. It is made available under a CC-BY 4.0 International license.

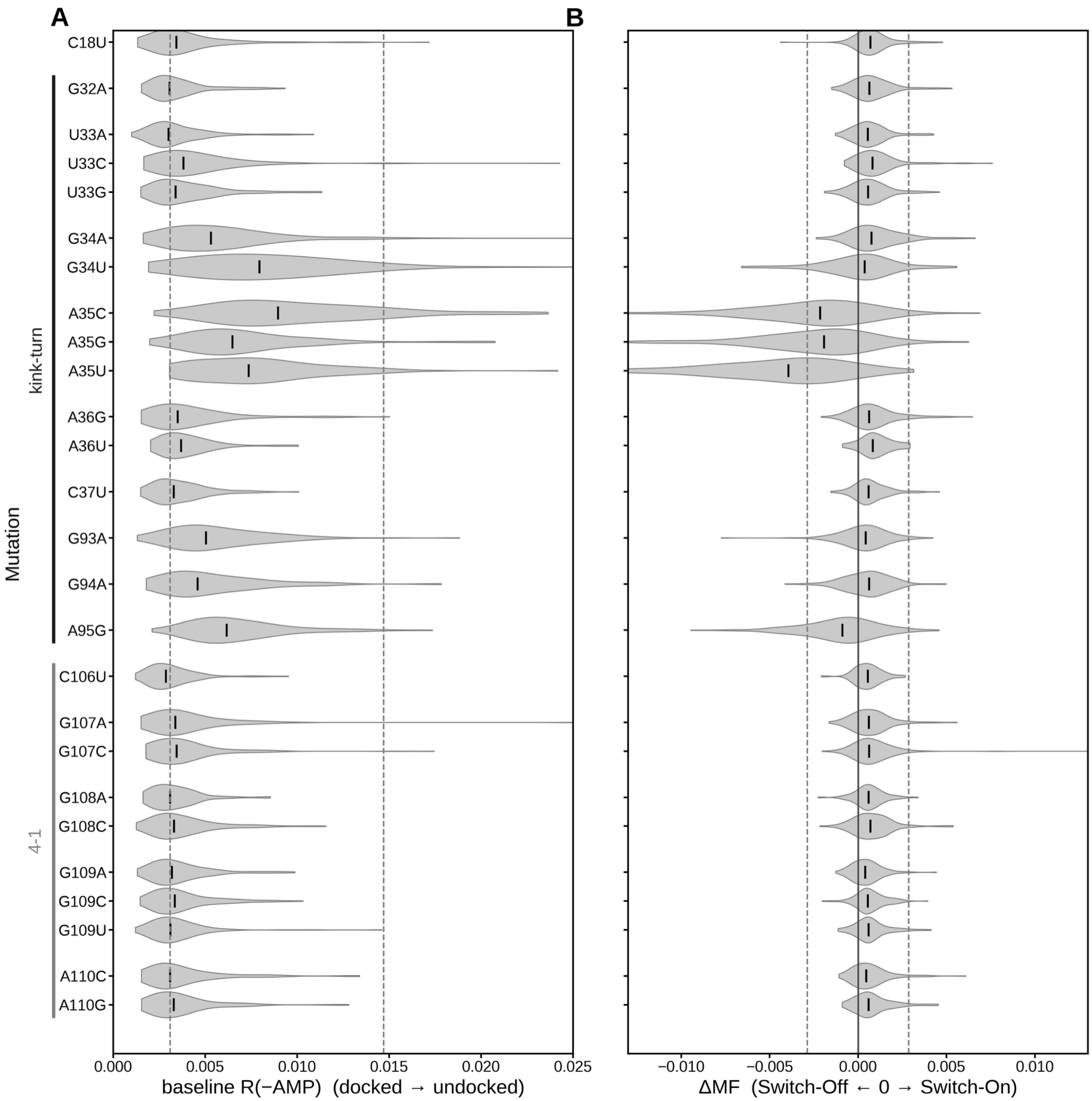

Figure S12: Junction single mutations shift the TL/TLR docking baseline and the AMP response by position

For each single junction mutation (rows, ordered along the construct and grouped by junction, kink-turn versus 4-1), the distribution across roughly 120 randomized helix backgrounds of (A) baseline TL/TLR reactivity without AMP, from docked (low) to undocked (high); the dashed lines are the θ_low and θ_high baseline cutoffs from Figure 2. (B) The AMP-induced change ΔMF(TL/TLR), where negative values are Switch-Off and positive values are Switch-On; the dashed lines are the ±3σ_helix cutoffs. The kink-turn 1b mutations A35C, A35G, and A35U shift

bioRxiv preprint doi: https://doi.org/10.64898/2026.08.09.743768; this version posted August 11, 2026. The copyright holder for this preprint (which was not certified by peer review) is the author/funder, who has granted bioRxiv a license to display the preprint in perpetuity. It is made available under a CC-BY 4.0 International license.

the baseline toward undocked and push ΔMF negative, and A95G behaves the same way, while the 4-1 junction mutations stay docked with near-zero ΔMF.

bioRxiv preprint doi: https://doi.org/10.64898/2026.08.09.743768; this version posted August 11, 2026. The copyright holder for this preprint (which was not certified by peer review) is the author/funder, who has granted bioRxiv a license to display the preprint in perpetuity. It is made available under a CC-BY 4.0 International license.

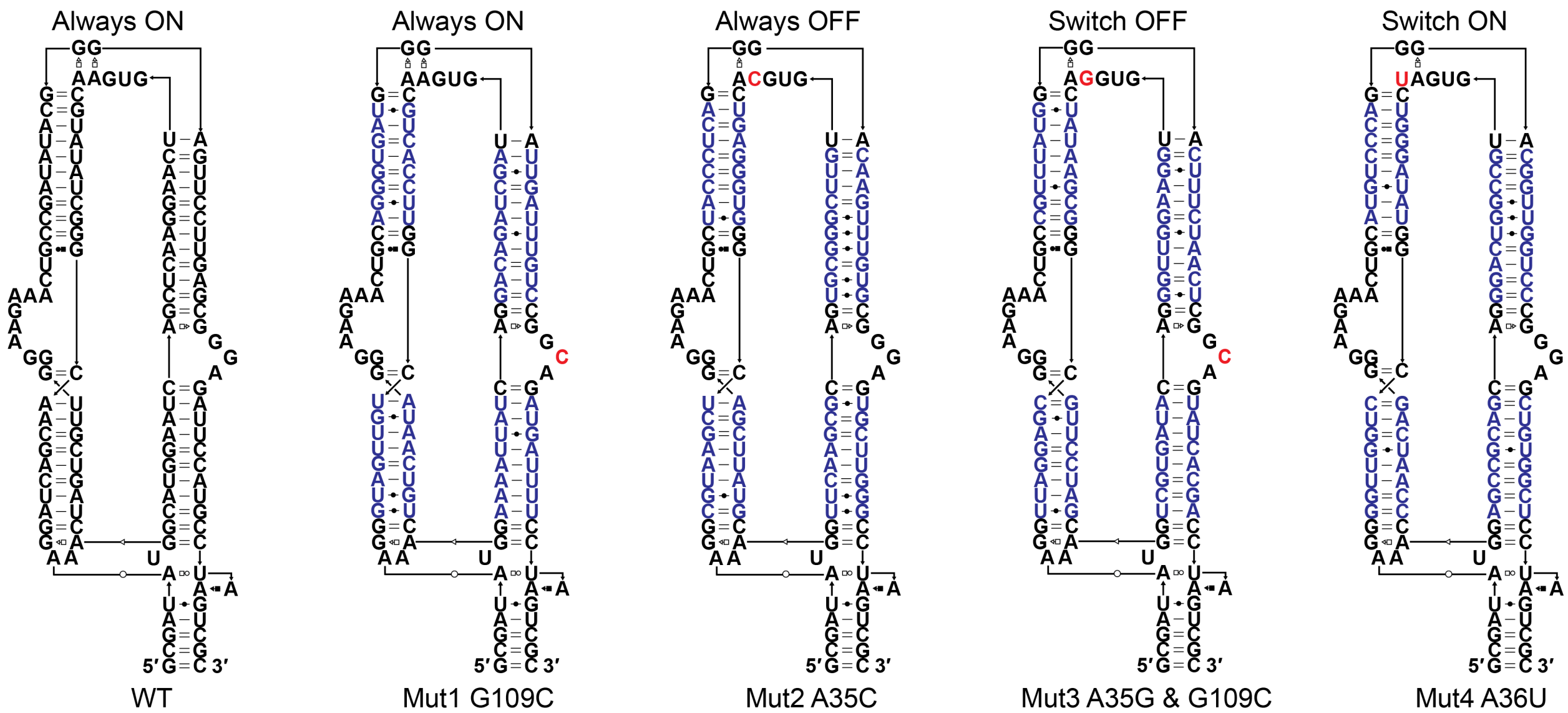

Figure S13: Secondary structures of the wild-type and the four representative mutants

Secondary-structure diagrams of the wild-type construct and the four phenotype representatives, labeled by allosteric regime (top) and by construct and mutation (bottom): wild-type (Always-On), Mut1 G109C (4-1 junction, Always-On), Mut2 A35C (kink-turn 1b, Always-Off), Mut3 A35G and G109C (kink-turn 1b plus 4-1, Switch-Off), and Mut4 A36U (kink-turn 2b, Switch-On). The mutated nucleotide is highlighted in red. Blue marks the helix-background positions that are randomized across library variants, drawn here as each representative's actual library sequence, while the wild-type reference is shown without randomization; black marks the fixed aptamer, junction, and TL/TLR positions.

bioRxiv preprint doi: https://doi.org/10.64898/2026.08.09.743768; this version posted August 11, 2026. The copyright holder for this preprint (which was not certified by peer review) is the author/funder, who has granted bioRxiv a license to display the preprint in perpetuity. It is made available under a CC-BY 4.0 International license.

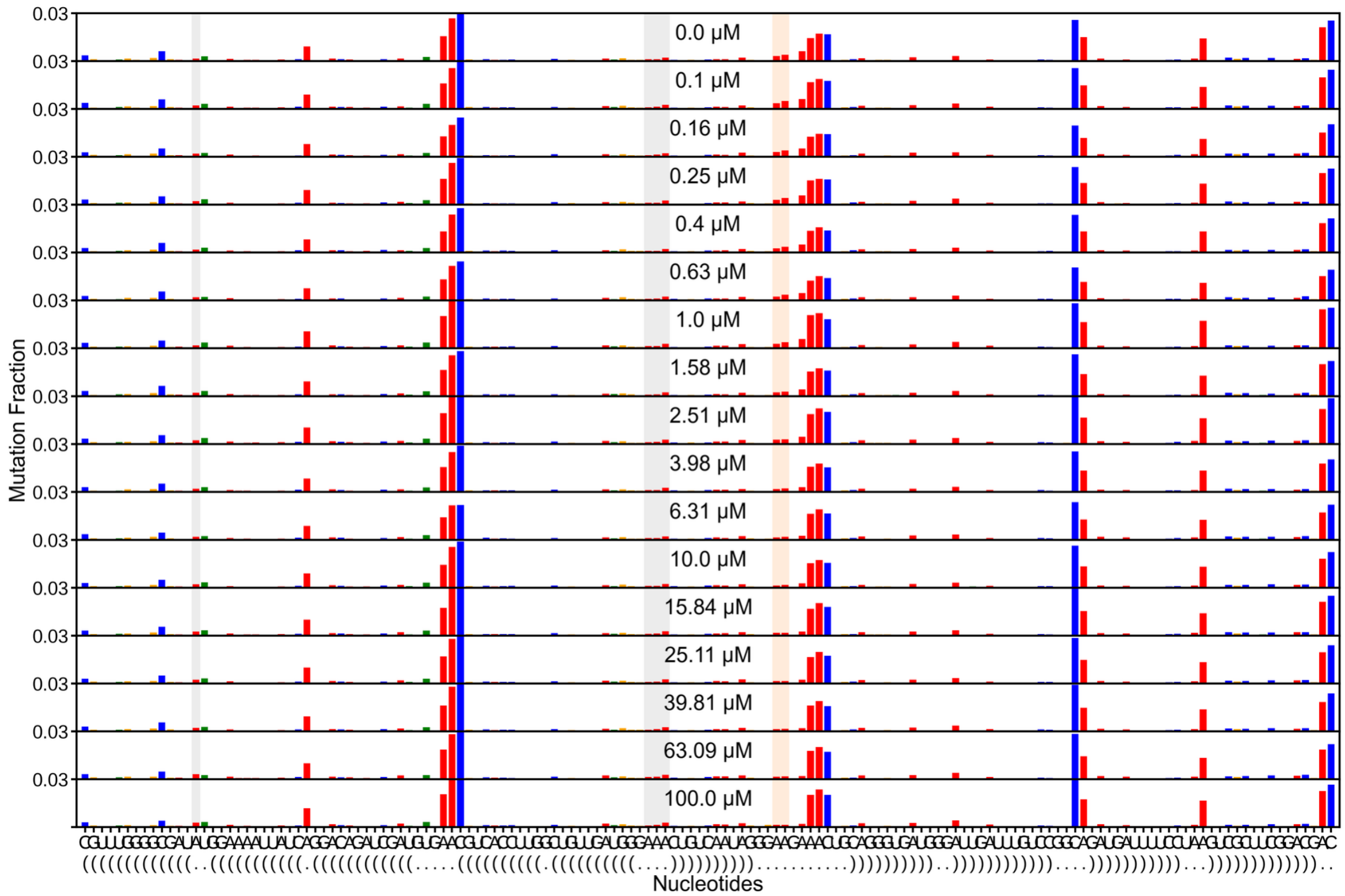

Figure S14: Always-On per-nucleotide reactivity across the AMP titration, replicate 1

Full per-nucleotide DMS-MaPseq reactivity for the Always-On representative (C01EI) at each point of the AMP titration (replicate 1), stacked by AMP concentration. Gray shading marks the TL/TLR docking reporters and orange shading the ATP-aptamer binding-site adenines. The aptamer adenines lose reactivity as AMP rises, so the aptamer binds AMP, while the TL/TLR reporters stay low across the titration, so the tertiary contact is docked regardless of ligand. These are the raw per-replicate data behind the Always-On curves in Figure 3B.

bioRxiv preprint doi: https://doi.org/10.64898/2026.08.09.743768; this version posted August 11, 2026. The copyright holder for this preprint (which was not certified by peer review) is the author/funder, who has granted bioRxiv a license to display the preprint in perpetuity. It is made available under a CC-BY 4.0 International license.

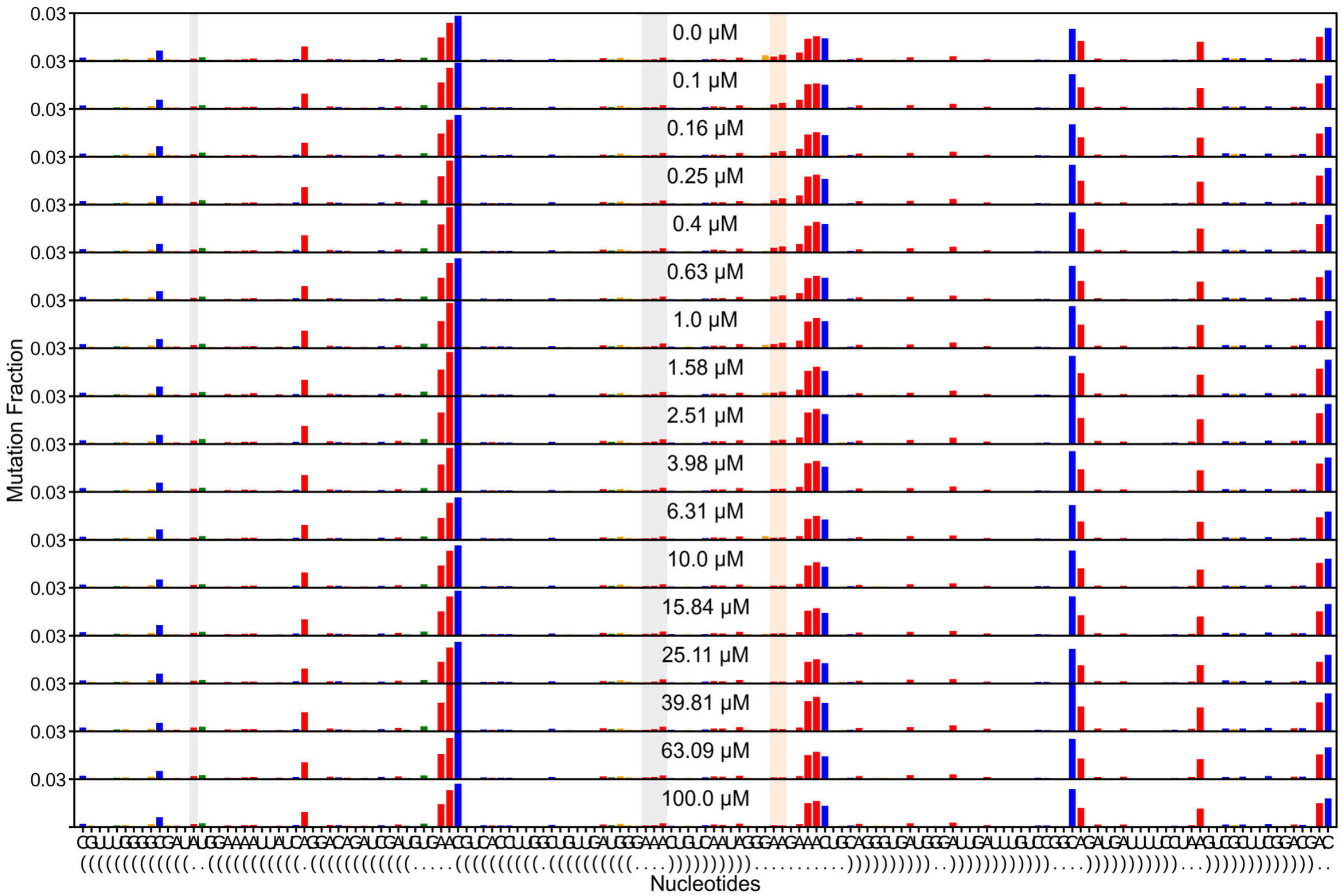

Figure S15: Always-On per-nucleotide reactivity across the AMP titration, replicate 2

As in Figure S13 for the second Always-On replicate. The aptamer adenines (orange) are protected as AMP increases while the TL/TLR reporters (gray) stay low, reproducing the docked, ligand-insensitive behavior of the Always-On regime.

bioRxiv preprint doi: https://doi.org/10.64898/2026.08.09.743768; this version posted August 11, 2026. The copyright holder for this preprint (which was not certified by peer review) is the author/funder, who has granted bioRxiv a license to display the preprint in perpetuity. It is made available under a CC-BY 4.0 International license.

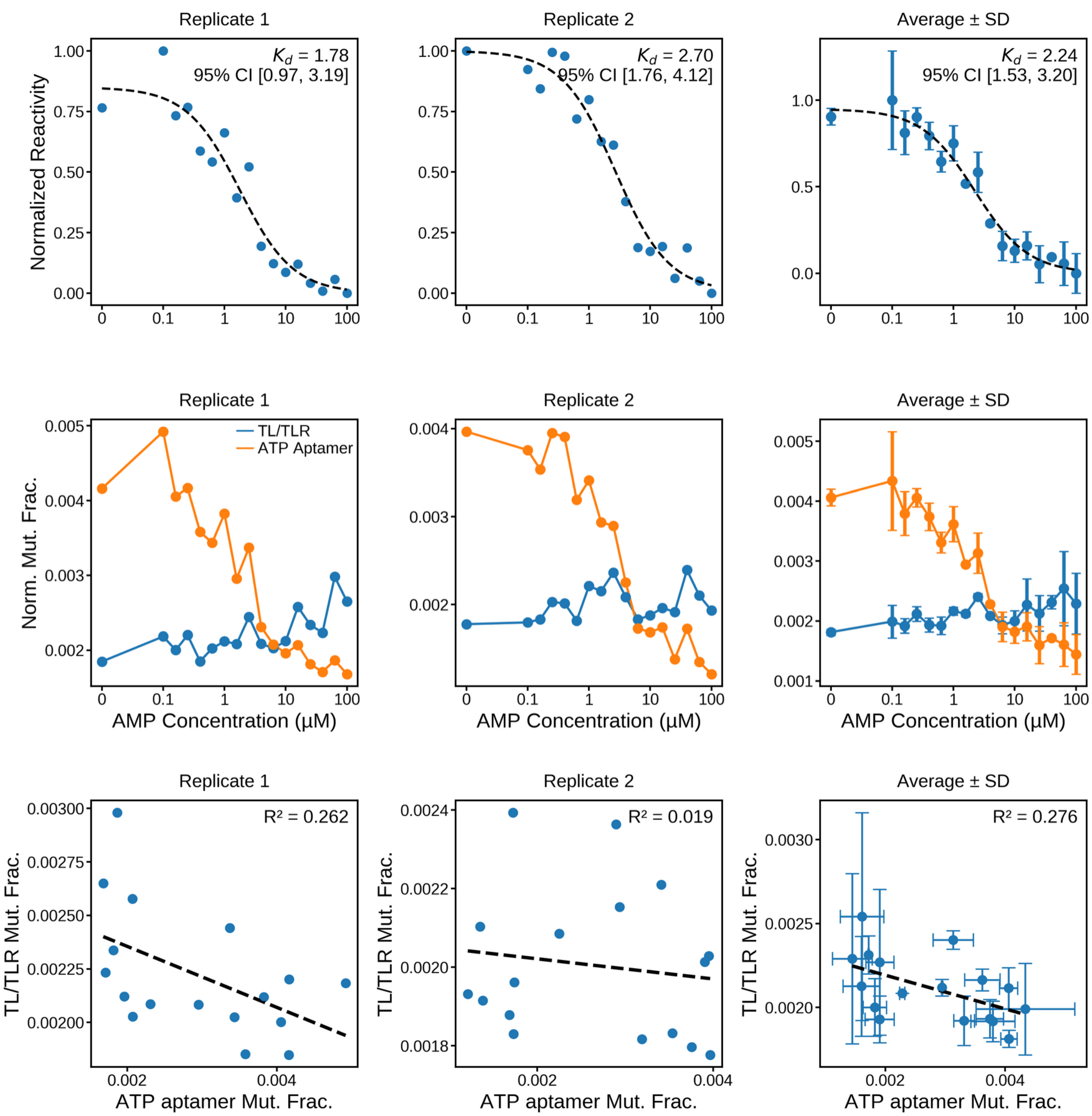

Figure S16: Always-On AMP binding affinity and weak coupling to TL/TLR docking

(Top) Mean reactivity of the AMP-sensitive ATP-aptamer binding-site adenines, normalized to [0, 1], versus AMP concentration for each Always-On replicate and for the inter-replicate average (± SD). Dashed lines are three-parameter Hill fits, with the apparent Kd annotated. The replicate fits give Kd = 1.78 ± 0.66 and 2.70 ± 0.60 µM, and averaging gives Kd = 2.24 ± 0.43 µM, so the Always-On representative binds AMP with wild-type-like affinity. (Middle) Per-nucleotide

bioRxiv preprint doi: https://doi.org/10.64898/2026.08.09.743768; this version posted August 11, 2026. The copyright holder for this preprint (which was not certified by peer review) is the author/funder, who has granted bioRxiv a license to display the preprint in perpetuity. It is made available under a CC-BY 4.0 International license.

normalized mutation fraction across the same titration for the TL/TLR docking reporters (blue) and the ATP-aptamer binding-site adenines (orange). Aptamer reactivity falls with AMP while the TL/TLR reporters stay low, so the contact stays docked throughout. (Bottom) TL/TLR docking reactivity versus ATP-aptamer reactivity across the titration, per replicate and averaged, with the coefficient of determination annotated.

bioRxiv preprint doi: https://doi.org/10.64898/2026.08.09.743768; this version posted August 11, 2026. The copyright holder for this preprint (which was not certified by peer review) is the author/funder, who has granted bioRxiv a license to display the preprint in perpetuity. It is made available under a CC-BY 4.0 International license.

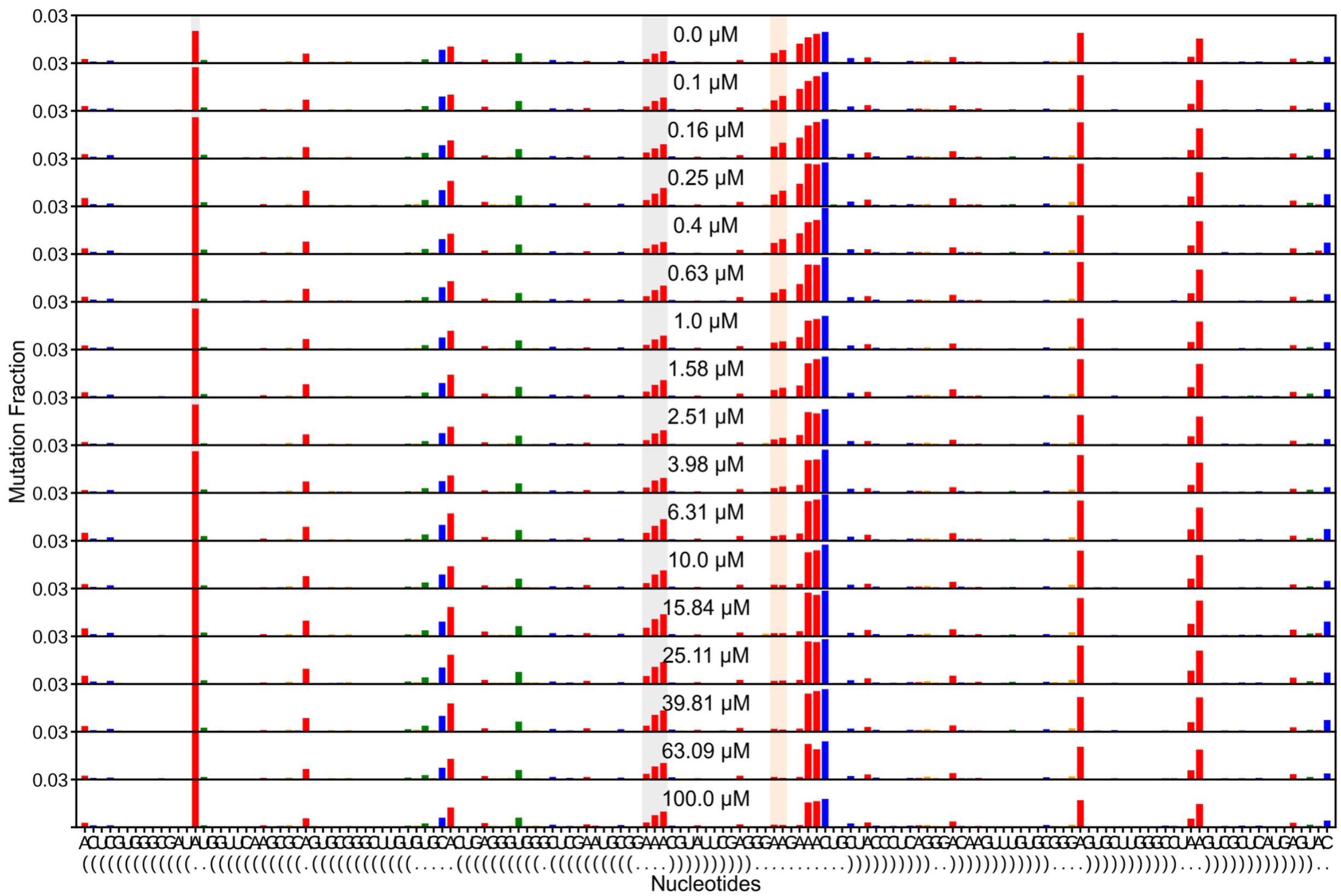

Figure S17: Always-Off per-nucleotide reactivity across the AMP titration, replicate 1

Full per-nucleotide DMS-MaPseq reactivity for the Always-Off representative (C01EK) at each point of the AMP titration (replicate 1), stacked from low AMP (top) to high AMP (bottom); each row is the mutation fraction at every nucleotide for one AMP concentration. Gray shading marks the TL/TLR docking reporters (the GAAA tetraloop adenines and the receptor adenine) and orange shading the ATP-aptamer binding-site adenines. The aptamer adenines lose reactivity as AMP rises, so the aptamer binds AMP, while the TL/TLR reporters stay high across the titration, so the tertiary contact is undocked regardless of ligand. These are the raw per-replicate data behind the Always-Off curves in Figure 3C.

bioRxiv preprint doi: https://doi.org/10.64898/2026.08.09.743768; this version posted August 11, 2026. The copyright holder for this preprint (which was not certified by peer review) is the author/funder, who has granted bioRxiv a license to display the preprint in perpetuity. It is made available under a CC-BY 4.0 International license.

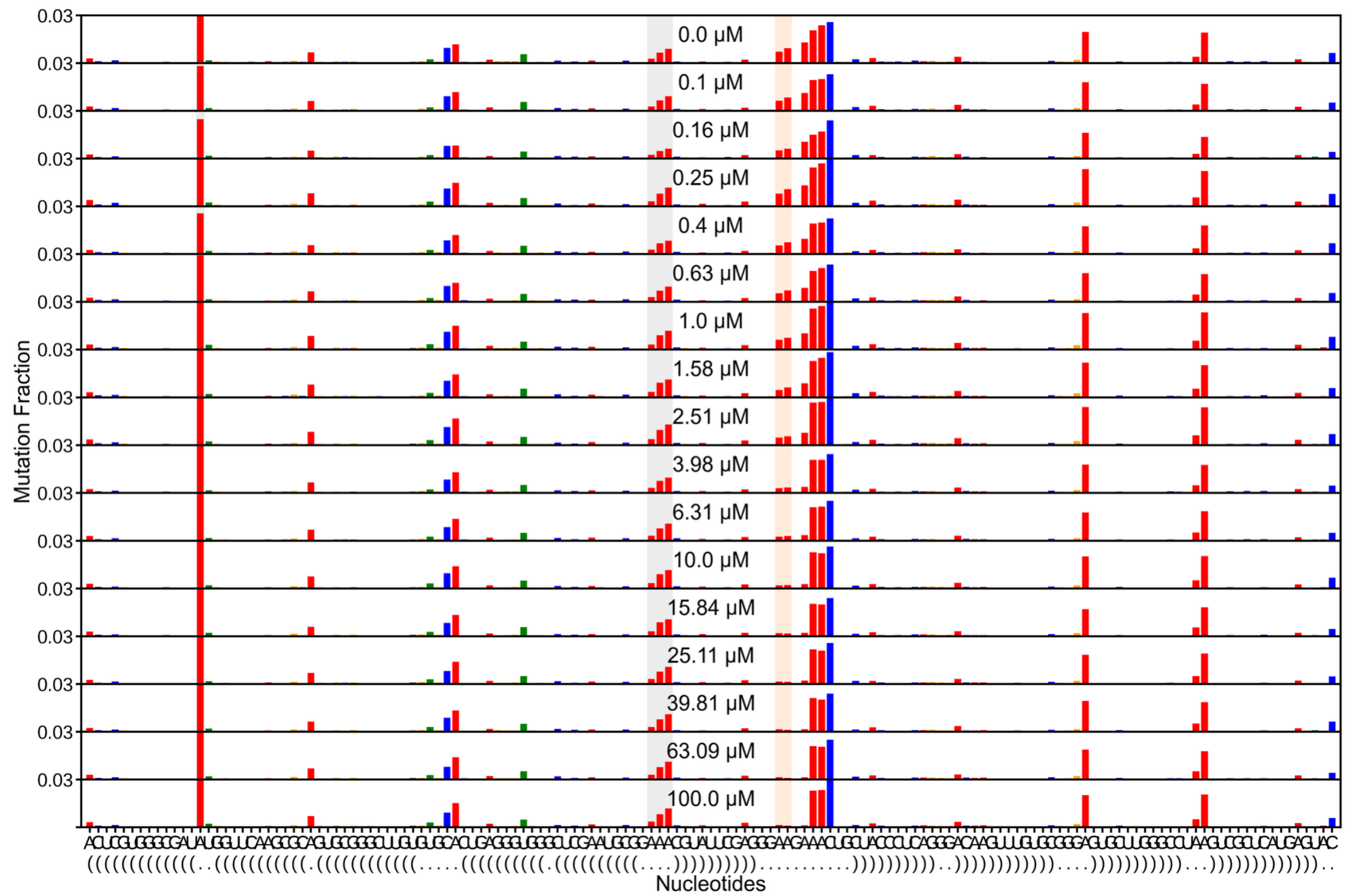

Figure S18: Always-Off per-nucleotide reactivity across the AMP titration, replicate 2

As in Figure S16 for the second Always-Off replicate. The aptamer adenines (orange) are protected as AMP increases while the TL/TLR reporters (gray) stay high, reproducing the undocked, ligand-insensitive docking of the Always-Off regime.

bioRxiv preprint doi: https://doi.org/10.64898/2026.08.09.743768; this version posted August 11, 2026. The copyright holder for this preprint (which was not certified by peer review) is the author/funder, who has granted bioRxiv a license to display the preprint in perpetuity. It is made available under a CC-BY 4.0 International license.

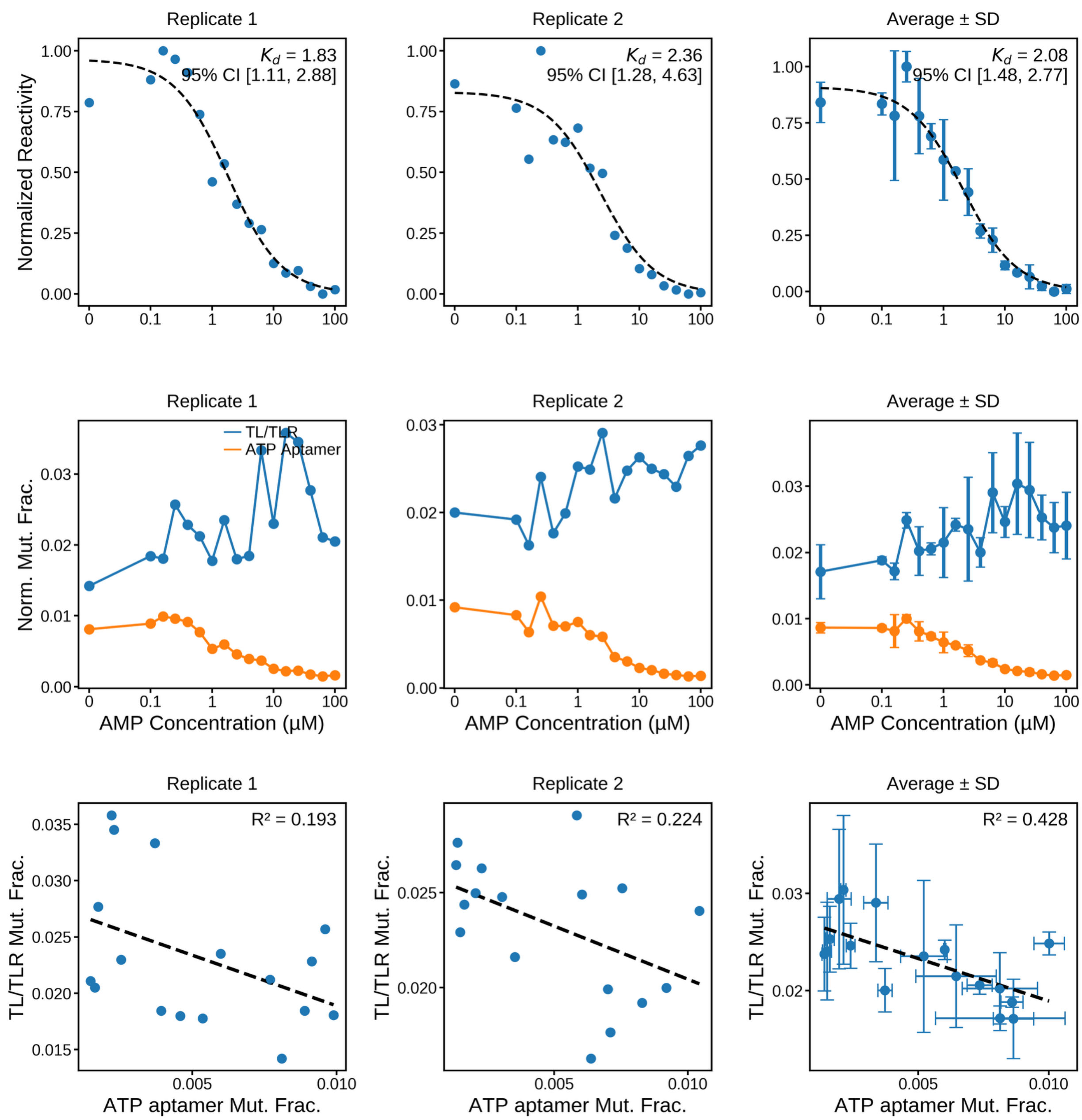

Figure S19: Always-Off AMP binding affinity and weak negative coupling to TL/TLR docking

(Top) Mean reactivity of the AMP-sensitive ATP-aptamer binding-site adenines, normalized to [0, 1], versus AMP concentration for each Always-Off replicate and for the inter-replicate average (± SD). Dashed lines are three-parameter Hill fits, with the apparent Kd annotated. The replicate fits give Kd = 1.83 ± 0.49 and 2.36 ± 0.76 μM, and averaging the replicates gives Kd = 2.08 ± 0.33 μM, so the Always-Off representative binds AMP with wild-type-like affinity. (Middle) Per-

bioRxiv preprint doi: https://doi.org/10.64898/2026.08.09.743768; this version posted August 11, 2026. The copyright holder for this preprint (which was not certified by peer review) is the author/funder, who has granted bioRxiv a license to display the preprint in perpetuity. It is made available under a CC-BY 4.0 International license.

nucleotide normalized mutation fraction across the same titration for the TL/TLR docking reporters (blue) and the ATP-aptamer binding-site adenines (orange). Aptamer reactivity falls with AMP, while the TL/TLR reporters stay high and rise modestly, so the contact is undocked with or without ligand. (Bottom) TL/TLR docking reactivity versus ATP-aptamer reactivity across the titration, per replicate and averaged, with the coefficient of determination annotated. The average anticorrelation (R^2 = 0.428) reflects the small AMP-induced rise in TL/TLR reactivity, the weak negative coupling quantified in Figure 3C.

bioRxiv preprint doi: https://doi.org/10.64898/2026.08.09.743768; this version posted August 11, 2026. The copyright holder for this preprint (which was not certified by peer review) is the author/funder, who has granted bioRxiv a license to display the preprint in perpetuity. It is made available under a CC-BY 4.0 International license.

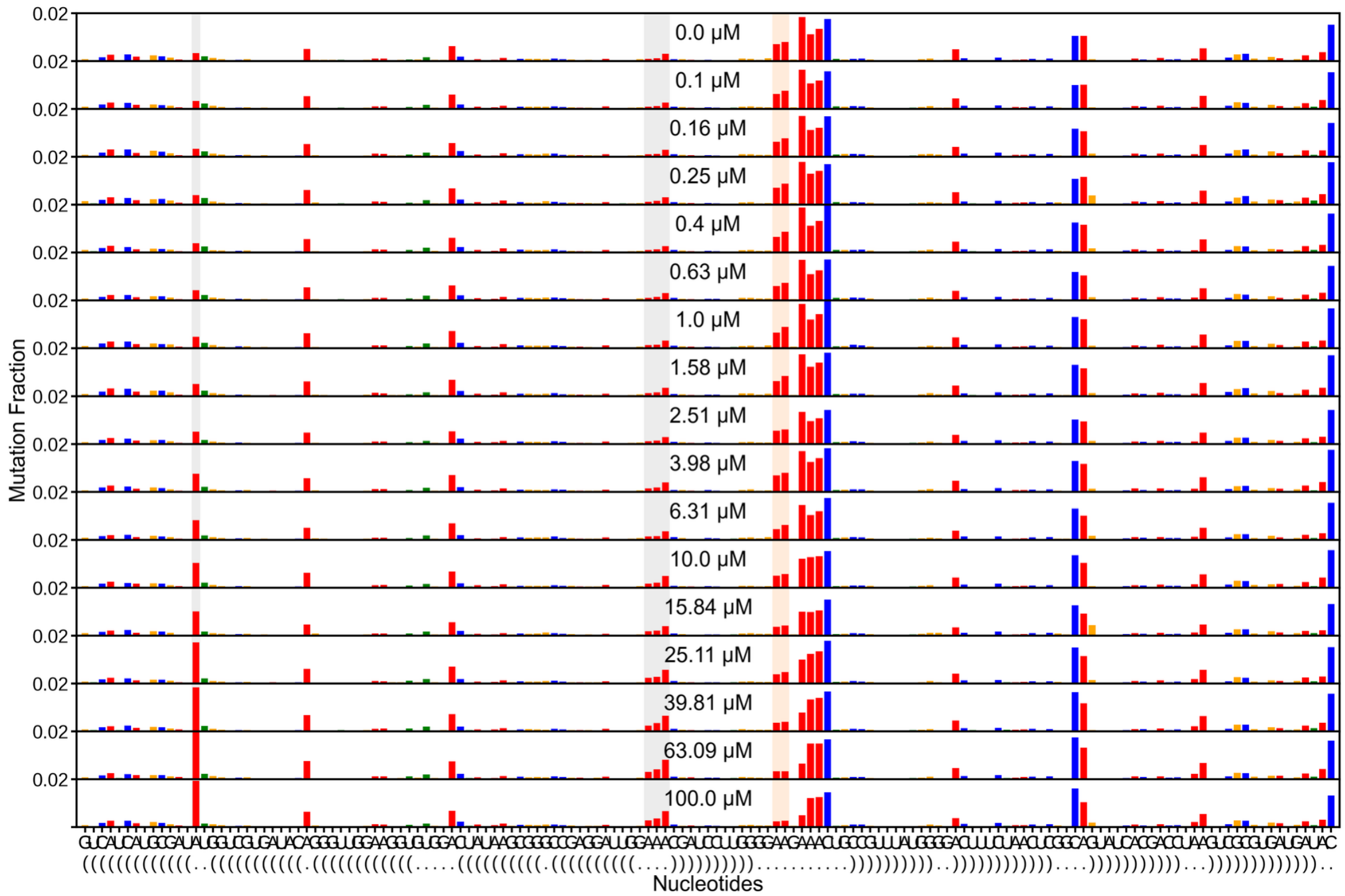

Figure S20: Switch-Off per-nucleotide reactivity across the AMP titration, replicate 1

Full per-nucleotide DMS-MaPseq reactivity for the Switch-Off representative (C01EJ) at each point of the AMP titration (replicate 1), stacked by AMP concentration. Gray shading marks the TL/TLR docking reporters and orange shading the ATP-aptamer binding-site adenines. The aptamer adenines lose reactivity as AMP rises, so the aptamer binds AMP, and the TL/TLR reporters gain reactivity over the same range, so AMP binding undocks the tertiary contact. These are the raw per-replicate data behind the Switch-Off curves in Figure 3D.

bioRxiv preprint doi: https://doi.org/10.64898/2026.08.09.743768; this version posted August 11, 2026. The copyright holder for this preprint (which was not certified by peer review) is the author/funder, who has granted bioRxiv a license to display the preprint in perpetuity. It is made available under a CC-BY 4.0 International license.

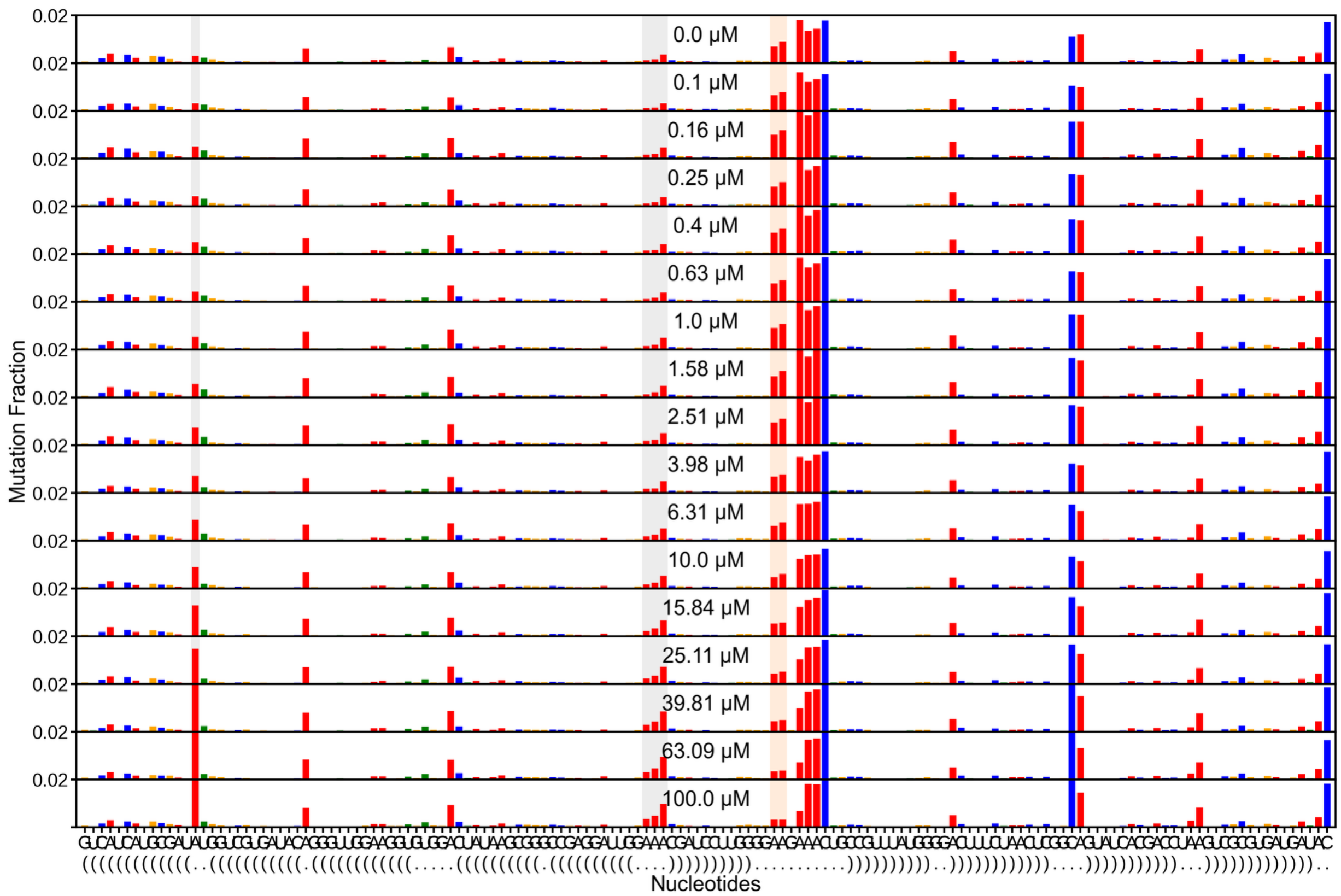

Figure S21: Switch-Off per-nucleotide reactivity across the AMP titration, replicate 2

As in Figure S19 for the second Switch-Off replicate. The aptamer adenines (orange) are protected while the TL/TLR reporters (gray) gain reactivity as AMP increases, reproducing the AMP-induced loss of docking that defines the Switch-Off regime.

bioRxiv preprint doi: https://doi.org/10.64898/2026.08.09.743768; this version posted August 11, 2026. The copyright holder for this preprint (which was not certified by peer review) is the author/funder, who has granted bioRxiv a license to display the preprint in perpetuity. It is made available under a CC-BY 4.0 International license.

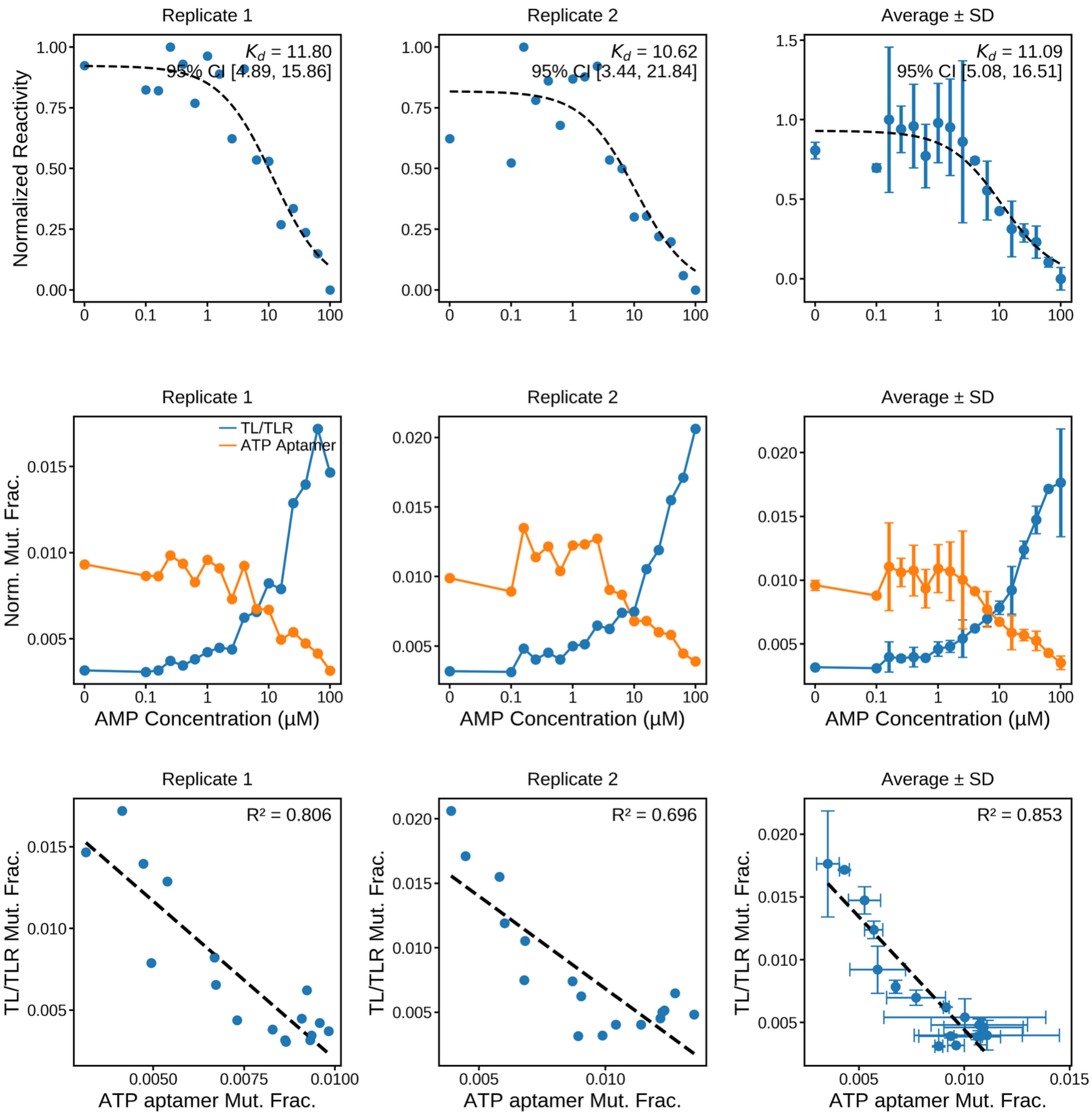

Figure S22: Switch-Off AMP binding drives loss of TL/TLR docking

(Top) Mean reactivity of the AMP-sensitive ATP-aptamer binding-site adenines, normalized to [0, 1], versus AMP concentration for each Switch-Off replicate and for the inter-replicate average (± SD). Dashed lines are three-parameter Hill fits, with the apparent Kd annotated. The replicate fits give Kd = 11.80 ± 2.98 and 10.62 ± 4.68 µM, and averaging gives Kd = 11.09 ± 2.99 µM. (Middle) Per-nucleotide normalized mutation fraction across the same titration for the TL/TLR docking reporters (blue) and the ATP-aptamer binding-site adenines (orange). The two arms move in opposite directions: aptamer reactivity falls as TL/TLR reactivity rises, so AMP binding undocks

bioRxiv preprint doi: https://doi.org/10.64898/2026.08.09.743768; this version posted August 11, 2026. The copyright holder for this preprint (which was not certified by peer review) is the author/funder, who has granted bioRxiv a license to display the preprint in perpetuity. It is made available under a CC-BY 4.0 International license.

the contact. (Bottom) TL/TLR docking reactivity versus ATP-aptamer reactivity across the titration, per replicate and averaged, with the coefficient of determination annotated. The strong average anticorrelation (R^2 = 0.853) reflects the tight AMP-coupled undocking, the negative coupling quantified in Figure 3D.

bioRxiv preprint doi: https://doi.org/10.64898/2026.08.09.743768; this version posted August 11, 2026. The copyright holder for this preprint (which was not certified by peer review) is the author/funder, who has granted bioRxiv a license to display the preprint in perpetuity. It is made available under a CC-BY 4.0 International license.

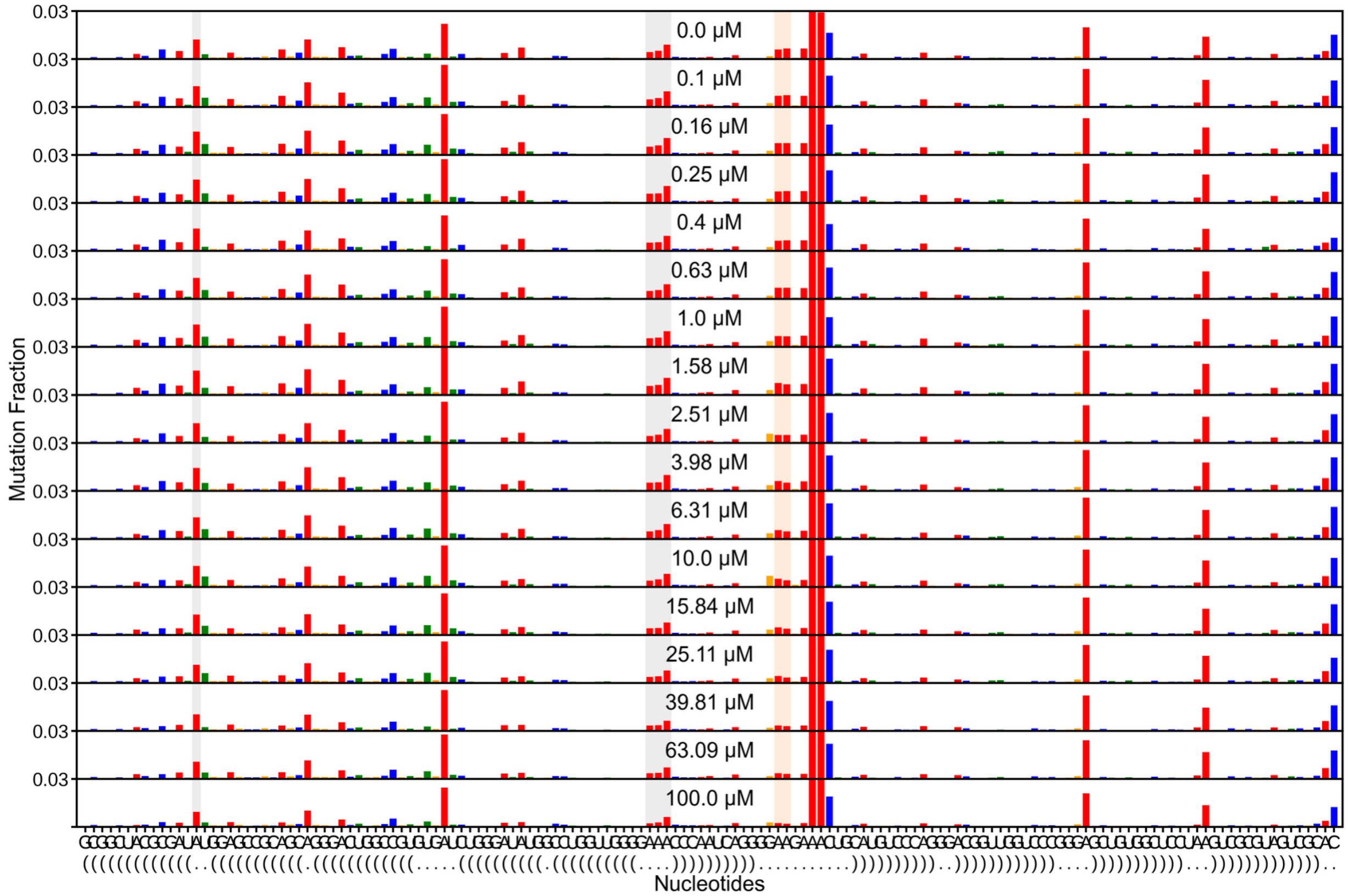

Figure S23: Switch-On per-nucleotide reactivity across the AMP titration at 22 °C, replicate 1

Full per-nucleotide DMS-MaPseq reactivity for the Switch-On representative (C01FL) at each point of the AMP titration (replicate 1), measured at 22 °C and stacked by AMP concentration. Gray shading marks the TL/TLR docking reporters and orange shading the ATP-aptamer binding-site adenines. The aptamer adenines lose reactivity as AMP rises, so AMP still binds at 22 °C, but the TL/TLR docking response is weaker and less consistent than at 44 °C (Figure S22). These are the raw per-replicate data behind the 22 °C fit in Figure S29.

bioRxiv preprint doi: https://doi.org/10.64898/2026.08.09.743768; this version posted August 11, 2026. The copyright holder for this preprint (which was not certified by peer review) is the author/funder, who has granted bioRxiv a license to display the preprint in perpetuity. It is made available under a CC-BY 4.0 International license.

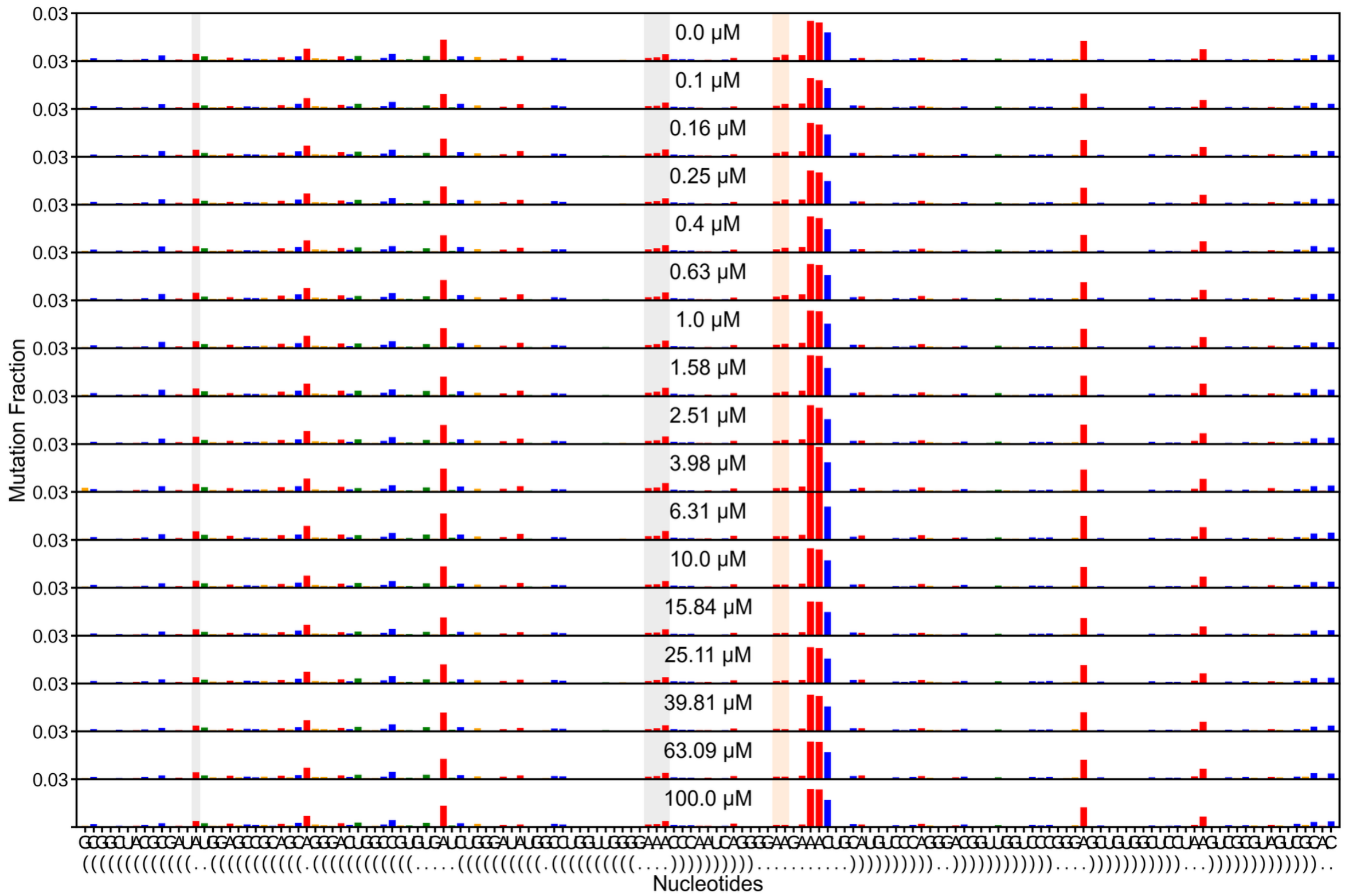

Figure S24: Switch-On per-nucleotide reactivity across the AMP titration at 22 °C, replicate 2

As in Figure S26 for the second 22 °C replicate. AMP protects the aptamer adenines (orange), but the TL/TLR reporters (blue) track binding only weakly, the replicate-variable docking response that motivated measuring Switch-On at 44 °C.

bioRxiv preprint doi: https://doi.org/10.64898/2026.08.09.743768; this version posted August 11, 2026. The copyright holder for this preprint (which was not certified by peer review) is the author/funder, who has granted bioRxiv a license to display the preprint in perpetuity. It is made available under a CC-BY 4.0 International license.

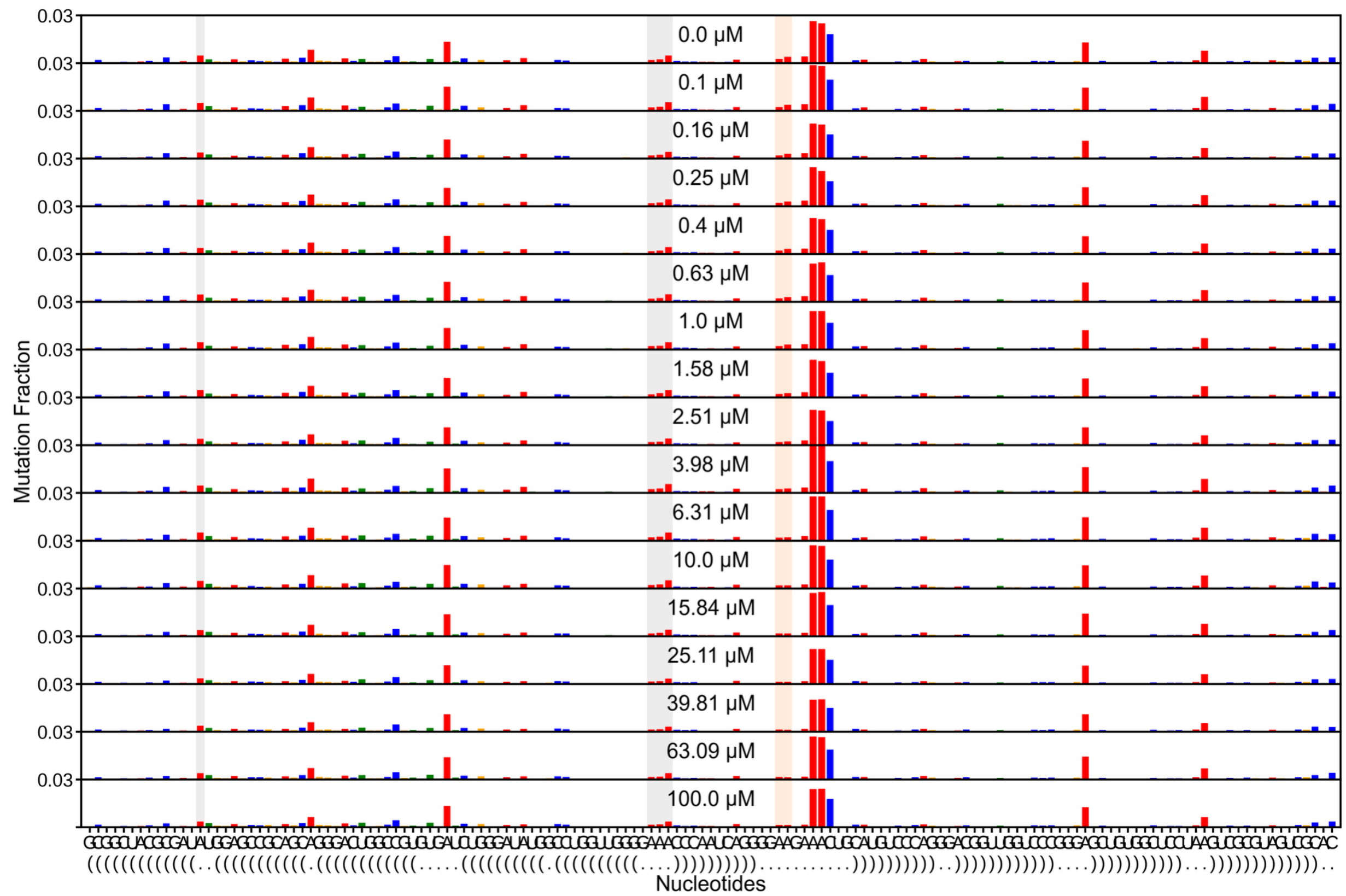

Figure S25: Switch-On per-nucleotide reactivity across the AMP titration at 22 °C, replicate 3

As in Figure S26 for the third 22 °C replicate. AMP again protects the aptamer adenines while the TL/TLR docking signal stays small, consistent with the attenuated 22 °C coupling.

bioRxiv preprint doi: https://doi.org/10.64898/2026.08.09.743768; this version posted August 11, 2026. The copyright holder for this preprint (which was not certified by peer review) is the author/funder, who has granted bioRxiv a license to display the preprint in perpetuity. It is made available under a CC-BY 4.0 International license.

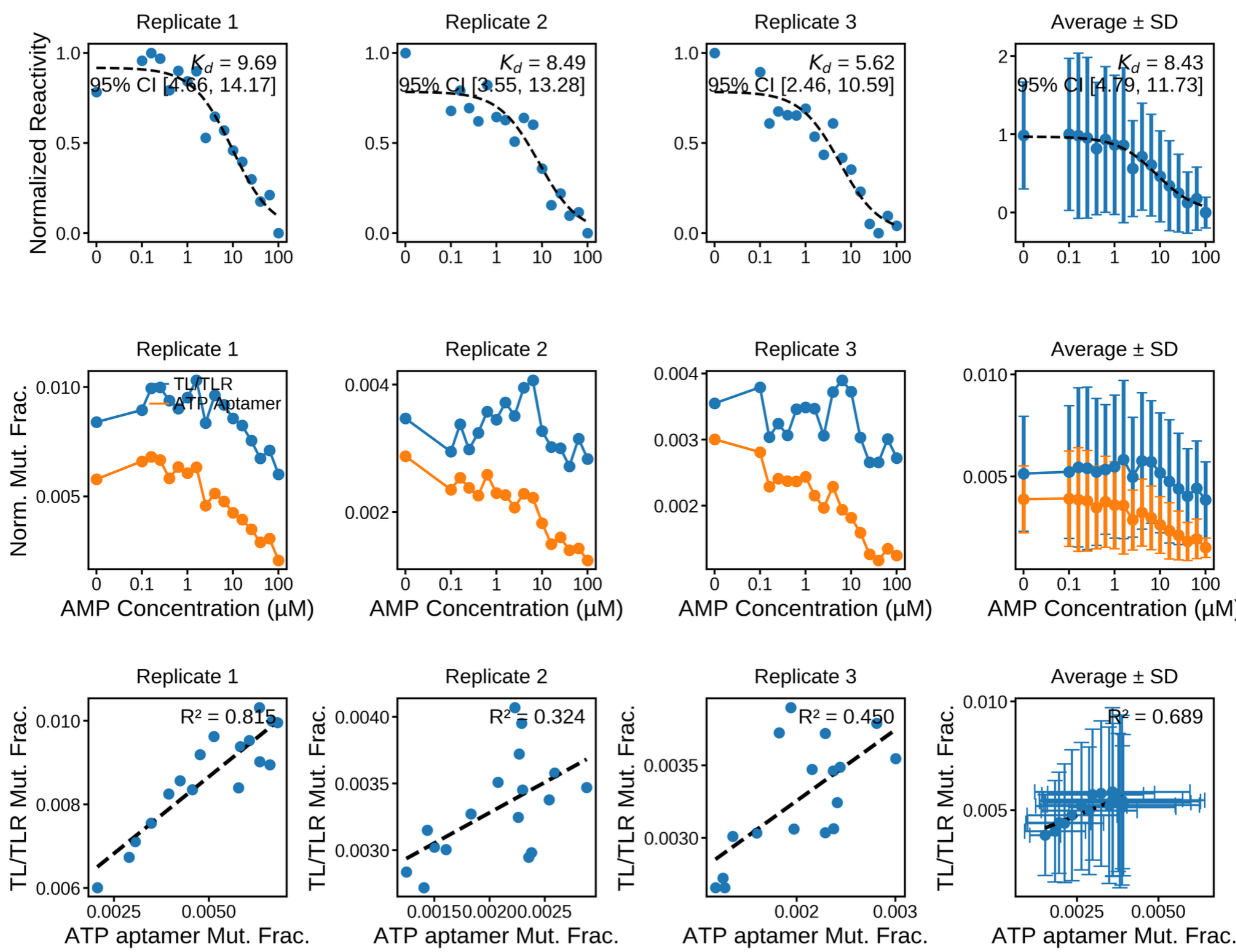

Figure S26: Switch-On AMP binding at 22 °C with attenuated TL/TLR docking

Measured at 22 °C over the same AMP range as the 44 °C panel (Figure S25). (Top) Mean reactivity of the AMP-sensitive ATP-aptamer binding-site adenines, normalized to [0, 1], versus AMP concentration for each replicate and the inter-replicate average (± SD). Dashed lines are three-parameter Hill fits, with the apparent Kd annotated. The replicate fits give Kd = 9.69 ± 2.40, 8.49 ± 2.78, and 5.62 ± 1.93 µM, and averaging gives Kd = 8.43 ± 1.57 µM, so the aptamer binds AMP at 22 °C. (Middle) Per-nucleotide normalized mutation fraction across the same titration for the TL/TLR docking reporters (blue) and the ATP-aptamer binding-site adenines (orange). The aptamer arm falls with AMP while the TL/TLR arm responds only weakly. (Bottom) TL/TLR docking reactivity versus ATP-aptamer reactivity across the titration, per replicate and averaged, with the coefficient of determination annotated. The correlation is weak and replicate-variable (R^2 = 0.815,

bioRxiv preprint doi: https://doi.org/10.64898/2026.08.09.743768; this version posted August 11, 2026. The copyright holder for this preprint (which was not certified by peer review) is the author/funder, who has granted bioRxiv a license to display the preprint in perpetuity. It is made available under a CC-BY 4.0 International license.

0.324, and 0.450; average 0.689), far below the 44 °C value (R^2 = 0.943, Figure S25). AMP binds at 22 °C but drives little docking; the same coupling amplifies into a reliable signal at 44 °C.

bioRxiv preprint doi: https://doi.org/10.64898/2026.08.09.743768; this version posted August 11, 2026. The copyright holder for this preprint (which was not certified by peer review) is the author/funder, who has granted bioRxiv a license to display the preprint in perpetuity. It is made available under a CC-BY 4.0 International license.

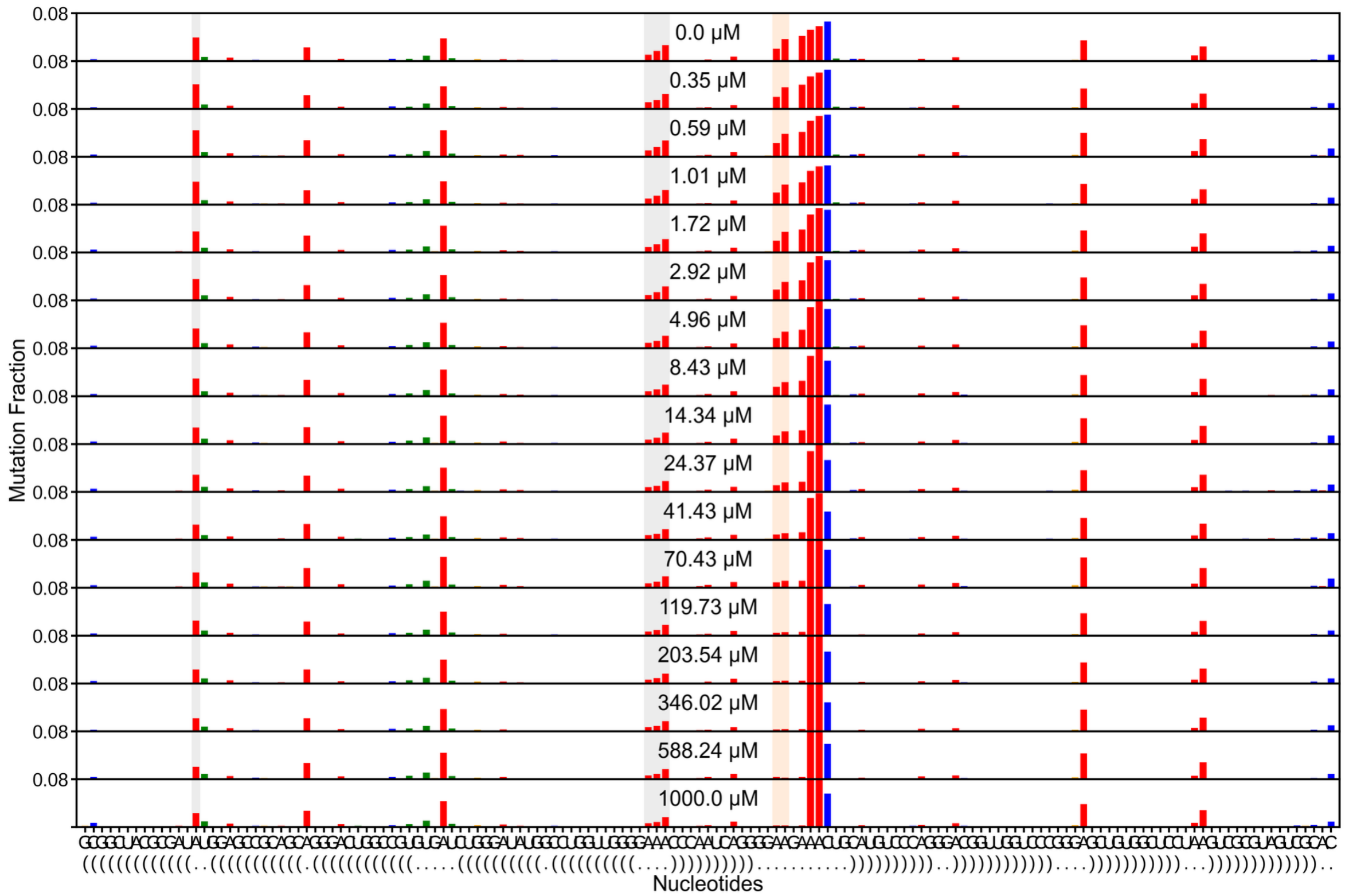

Figure S27: Switch-On per-nucleotide reactivity across the AMP titration, replicate 1

Full per-nucleotide DMS-MaPseq reactivity for the Switch-On representative (C01FL) at each point of the AMP titration (replicate 1), measured at 44 °C over a wider AMP range (to 1000 µM) and stacked by AMP concentration. Gray shading marks the TL/TLR docking reporters and orange shading the ATP-aptamer binding-site adenines. Both sets of reporters lose reactivity as AMP rises: the aptamer adenines report binding and the TL/TLR reporters report docking, so AMP binding drives the tertiary contact to dock. These are the raw per-replicate data behind the Switch-On curves in Figure 3E.

bioRxiv preprint doi: https://doi.org/10.64898/2026.08.09.743768; this version posted August 11, 2026. The copyright holder for this preprint (which was not certified by peer review) is the author/funder, who has granted bioRxiv a license to display the preprint in perpetuity. It is made available under a CC-BY 4.0 International license.

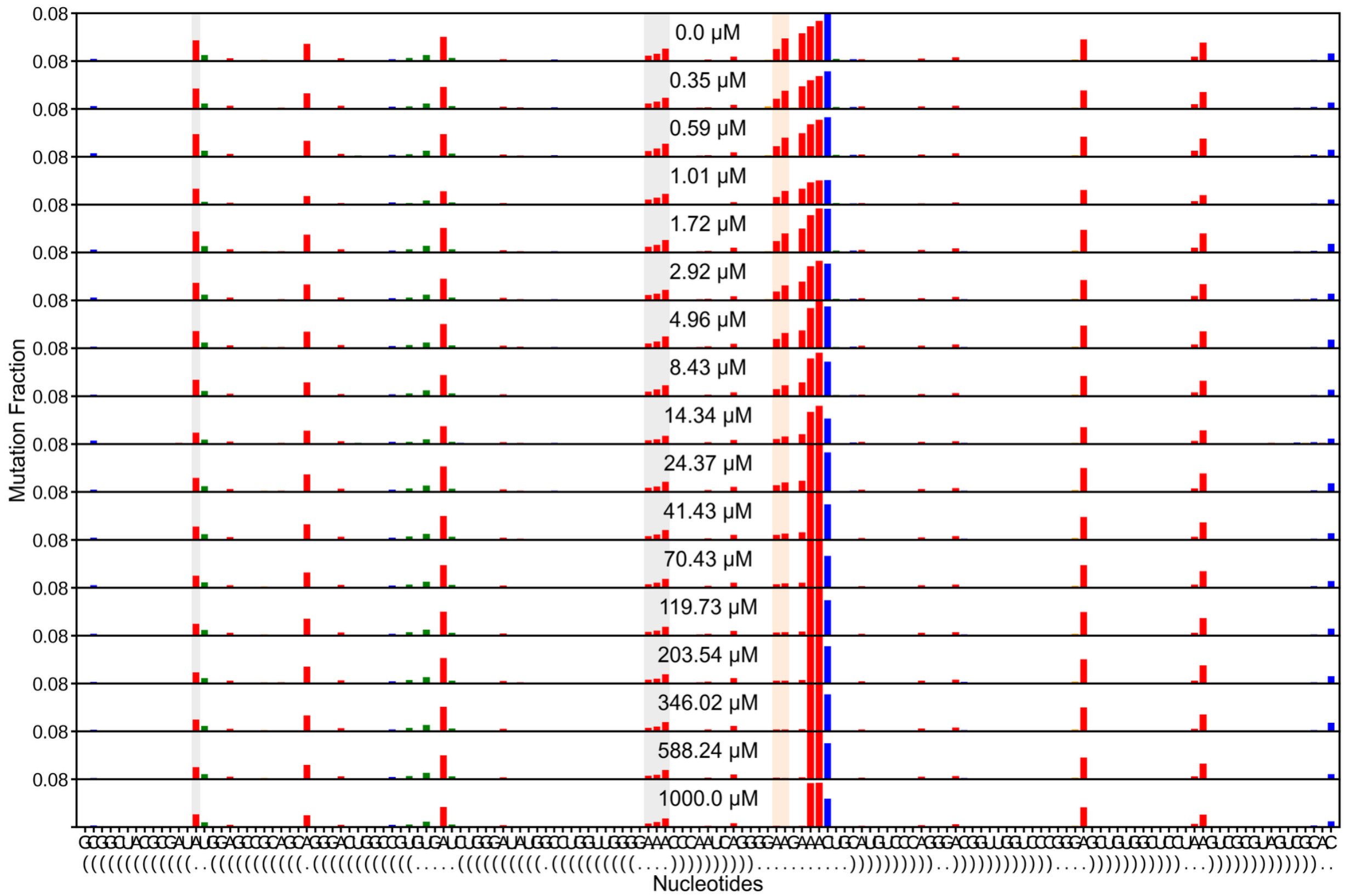

Figure S28: Switch-On per-nucleotide reactivity across the AMP titration, replicate 2

As in Figure S22 for the second Switch-On replicate. Both the aptamer (orange) and TL/TLR (gray) reporters are protected as AMP increases, reproducing the AMP-induced gain of docking that defines the Switch-On regime.

bioRxiv preprint doi: https://doi.org/10.64898/2026.08.09.743768; this version posted August 11, 2026. The copyright holder for this preprint (which was not certified by peer review) is the author/funder, who has granted bioRxiv a license to display the preprint in perpetuity. It is made available under a CC-BY 4.0 International license.

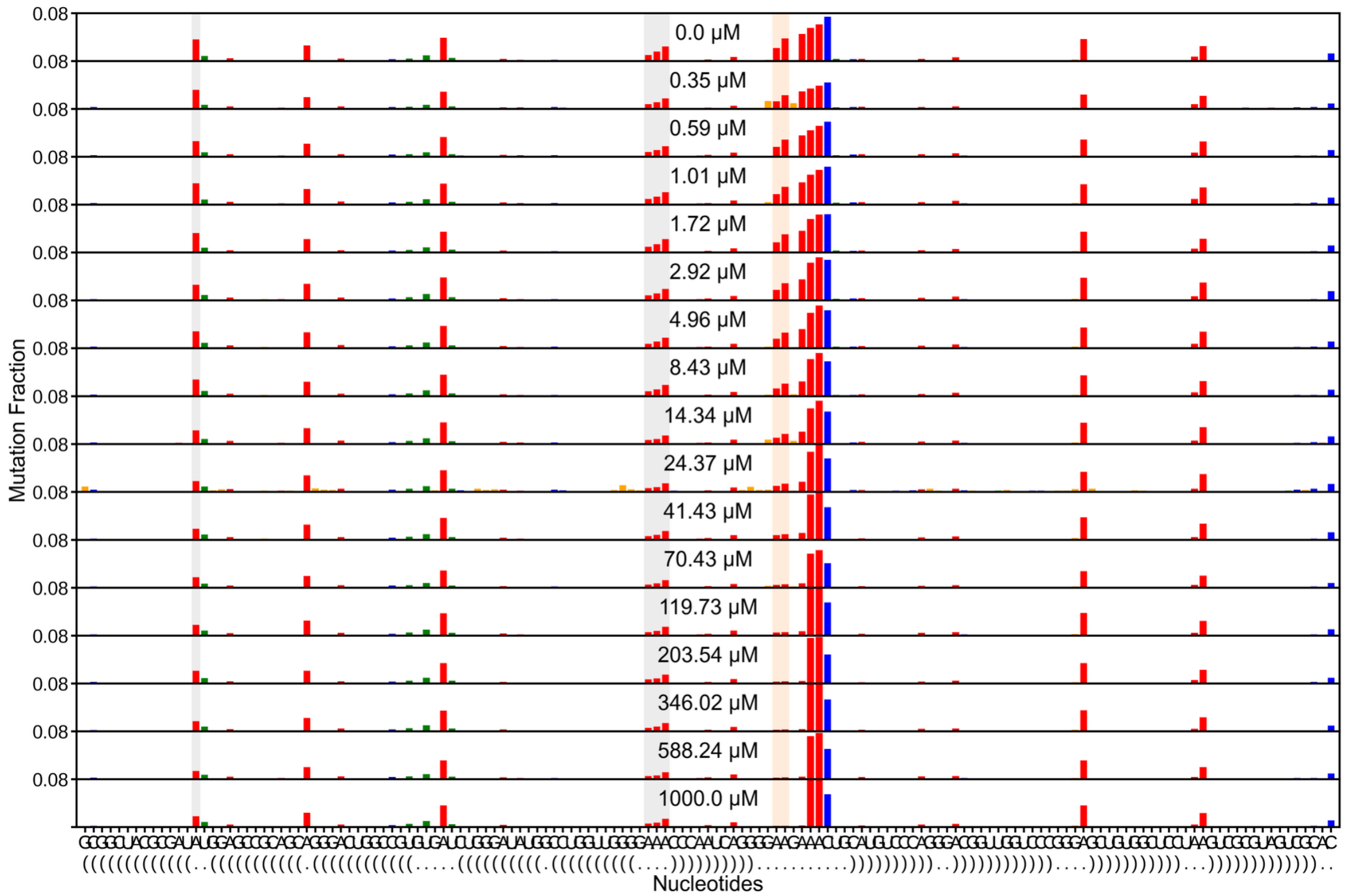

Figure S29: Switch-On per-nucleotide reactivity across the AMP titration, replicate 3

As in Figure S22 for the third Switch-On replicate. The aptamer and TL/TLR reporters lose reactivity together as AMP increases, consistent across all three replicates.

bioRxiv preprint doi: https://doi.org/10.64898/2026.08.09.743768; this version posted August 11, 2026. The copyright holder for this preprint (which was not certified by peer review) is the author/funder, who has granted bioRxiv a license to display the preprint in perpetuity. It is made available under a CC-BY 4.0 International license.

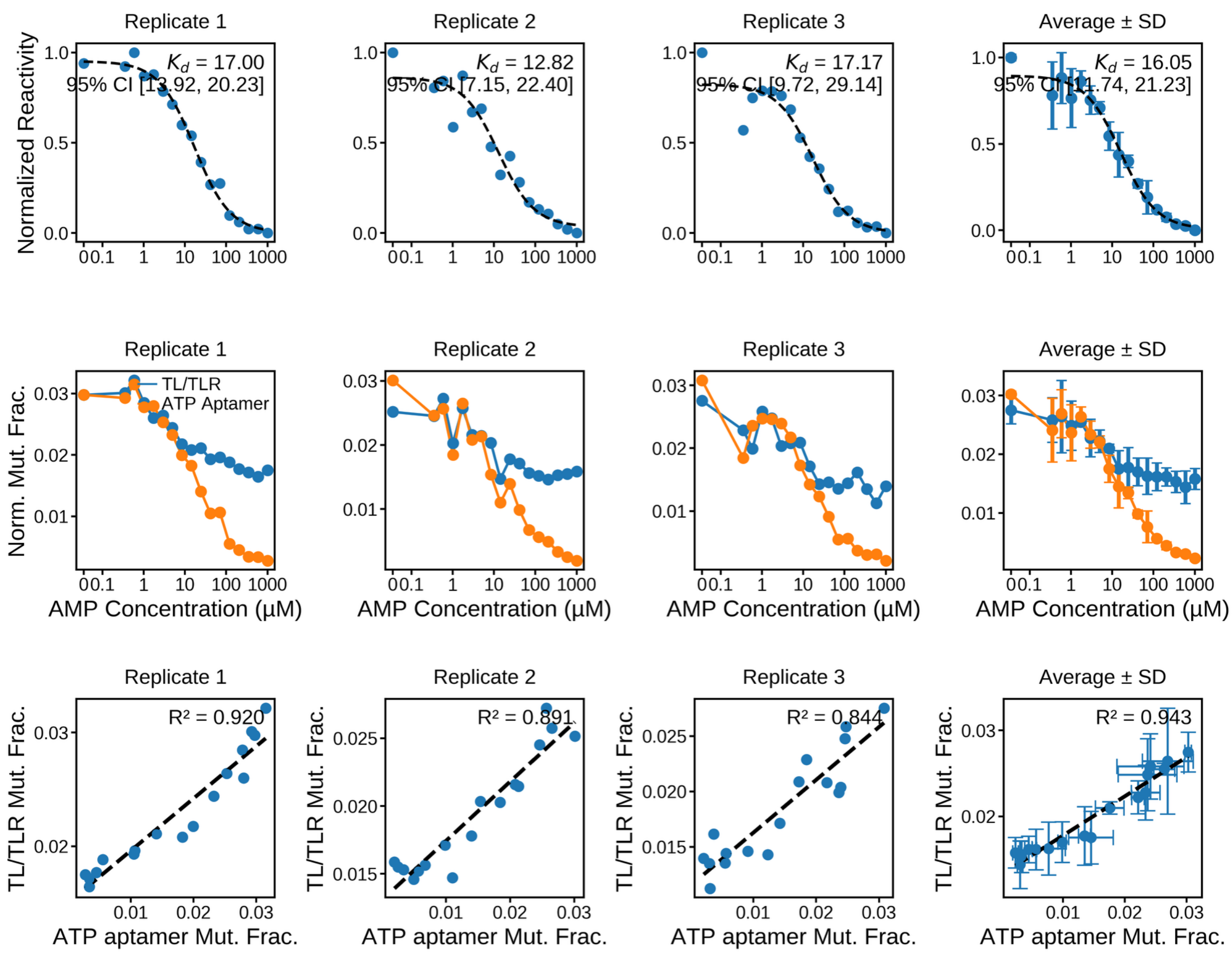

Figure S30: Switch-On AMP binding drives TL/TLR docking

Measured at 44 °C over a wider AMP range (to 1000 μM). (Top) Mean reactivity of the AMP-sensitive ATP-aptamer binding-site adenines, normalized to [0, 1], versus AMP concentration for each Switch-On replicate and for the inter-replicate average (± SD). Dashed lines are three-parameter Hill fits, with the apparent Kd annotated. The replicate fits give Kd = 17.00 ± 1.74, 12.82 ± 3.80, and 17.17 ± 5.01 μM, and averaging gives Kd = 16.05 ± 2.54 μM. (Middle) Per-nucleotide normalized mutation fraction across the same titration for the TL/TLR docking reporters (blue) and the ATP-aptamer binding-site adenines (orange). Both arms fall together as AMP rises, so AMP binding and TL/TLR docking track each other. (Bottom) TL/TLR docking reactivity versus ATP-aptamer reactivity across the titration, per replicate and averaged, with the coefficient of determination annotated. The strong average correlation (R^2 = 0.943) reflects the tight AMP-coupled docking, the positive coupling quantified in Figure 3E.

bioRxiv preprint doi: https://doi.org/10.64898/2026.08.09.743768; this version posted August 11, 2026. The copyright holder for this preprint (which was not certified by peer review) is the author/funder, who has granted bioRxiv a license to display the preprint in perpetuity. It is made available under a CC-BY 4.0 International license.

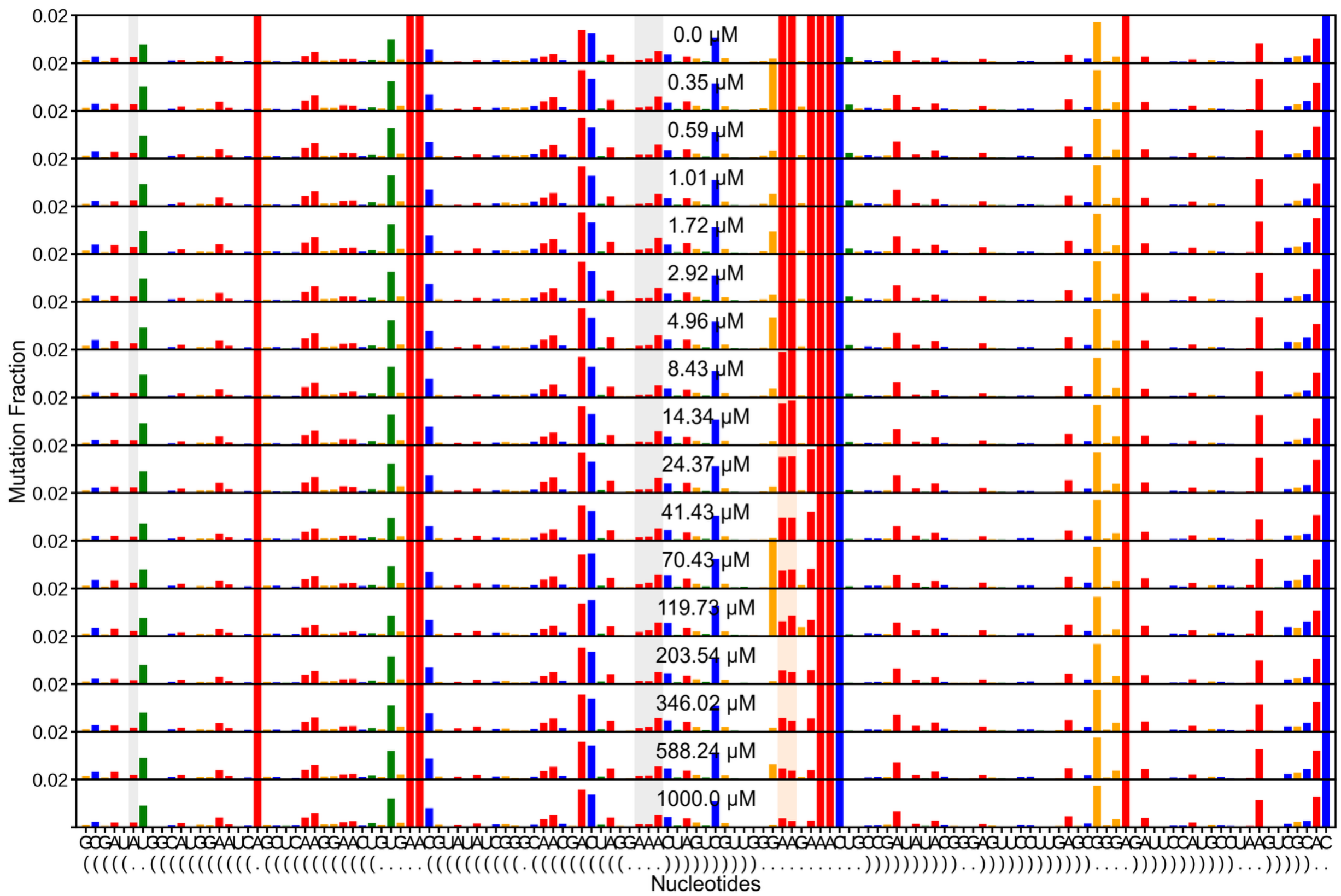

Figure S31: WT AMP titration at 44 °C, full per-nucleotide reactivity

Stacked population-average per-nucleotide reactivity across the AMP titration for the wild type at 44 °C (run 67, single replicate; n = 1). The y-axis is fixed to 0.02, the scale used for the 22 °C version (Figure S1), so the two temperatures compare directly. As at 22 °C, the TL/TLR reporters stay low across the titration, so the wild type remains docked at 44 °C.

bioRxiv preprint doi: https://doi.org/10.64898/2026.08.09.743768; this version posted August 11, 2026. The copyright holder for this preprint (which was not certified by peer review) is the author/funder, who has granted bioRxiv a license to display the preprint in perpetuity. It is made available under a CC-BY 4.0 International license.

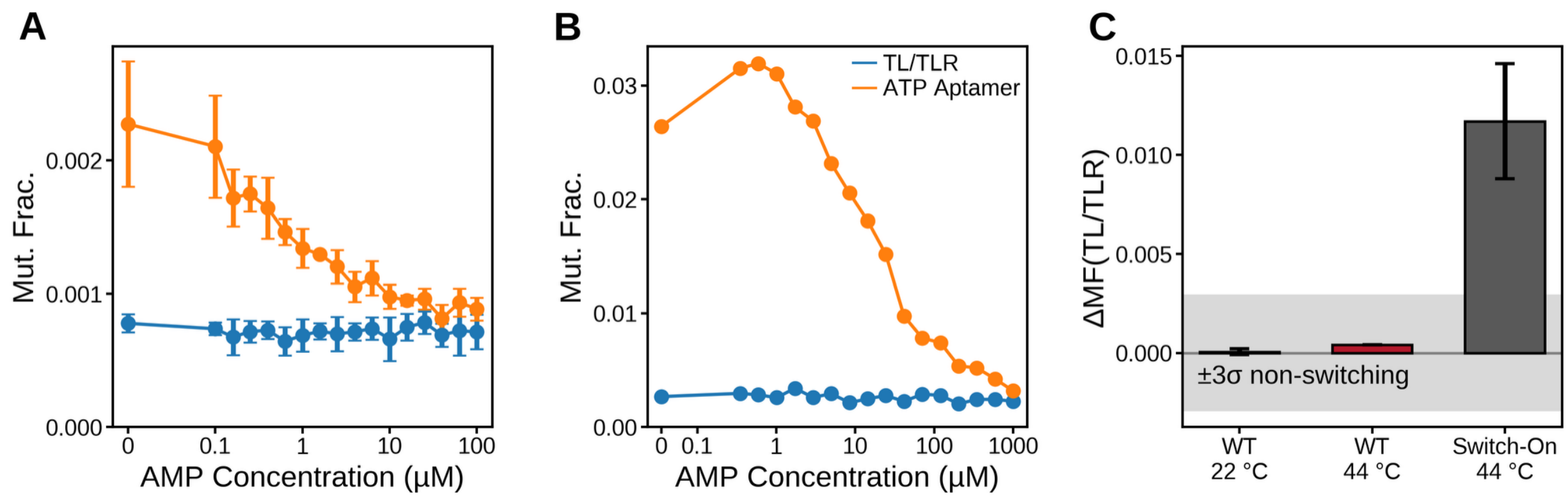

Figure S32: The wild type holds its regime at 44 °C

The Switch-On representative was measured at 44 °C, so this figure tests whether 44 °C by itself changes the wild-type regime. Dual AMP titration at (A) 22 °C (mean ± SD over replicates 2 to 4) and (B) 44 °C (single replicate from run 67; n = 1, no error bars), with the TL/TLR docking reporter (blue) and the ATP-aptamer reporter (orange). At both temperatures, the aptamer arm falls as AMP binds, while the TL/TLR arm stays flat. (C) ΔMF(TL/TLR), the TL/TLR reactivity without AMP minus that at saturating AMP, the same quantity used to classify the screen, against the ±3σ non-switching band (σ from the helix-only null distribution). Absolute DMS reactivity rises with temperature, so the regime is judged on this difference rather than on raw level. The wild type falls inside the band at both temperatures, while the Switch-On representative at 44 °C sits four times outside it.

bioRxiv preprint doi: https://doi.org/10.64898/2026.08.09.743768; this version posted August 11, 2026. The copyright holder for this preprint (which was not certified by peer review) is the author/funder, who has granted bioRxiv a license to display the preprint in perpetuity. It is made available under a CC-BY 4.0 International license.

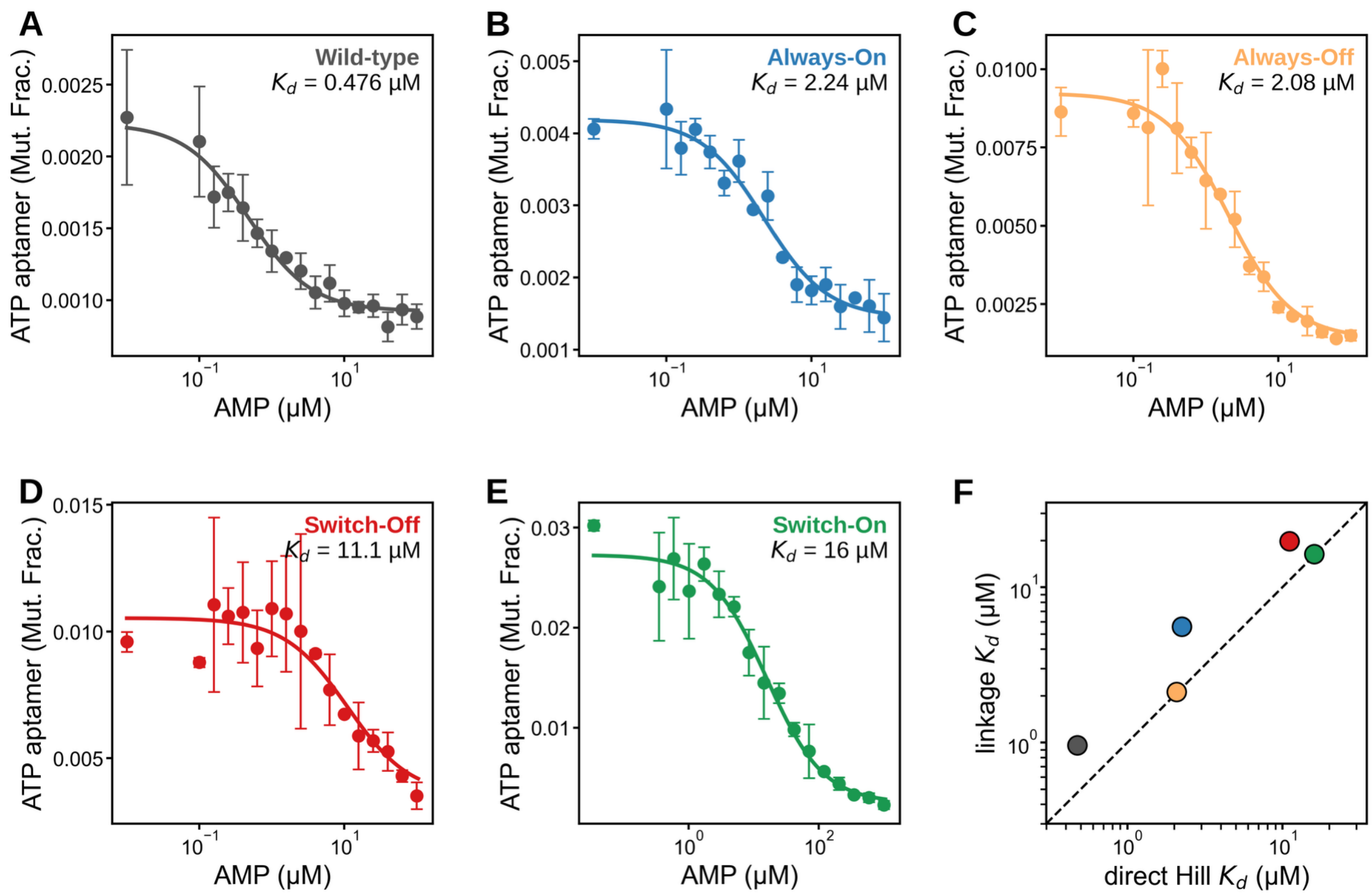

Figure S33: AMP affinity from direct Hill fits and Kd comparison

(A to E) The ATP-aptamer AMP titration for the wild type and the four representatives, each fit to a three-parameter Hill equation with the apparent K_d annotated, plotted on a log AMP axis in the class colors of Figure 4. Switch-On is measured at 44 °C. (F) Apparent AMP Kd from the direct Hill fit (x-axis) versus the four-state linkage-model K_d (y-axis). The two agree where the TL/TLR arm responds to AMP and diverge for the flat-arm constructs (wild type, Always-On), where the linkage K_d is underdetermined; the direct Hill value is reported for those cases.

bioRxiv preprint doi: https://doi.org/10.64898/2026.08.09.743768; this version posted August 11, 2026. The copyright holder for this preprint (which was not certified by peer review) is the author/funder, who has granted bioRxiv a license to display the preprint in perpetuity. It is made available under a CC-BY 4.0 International license.

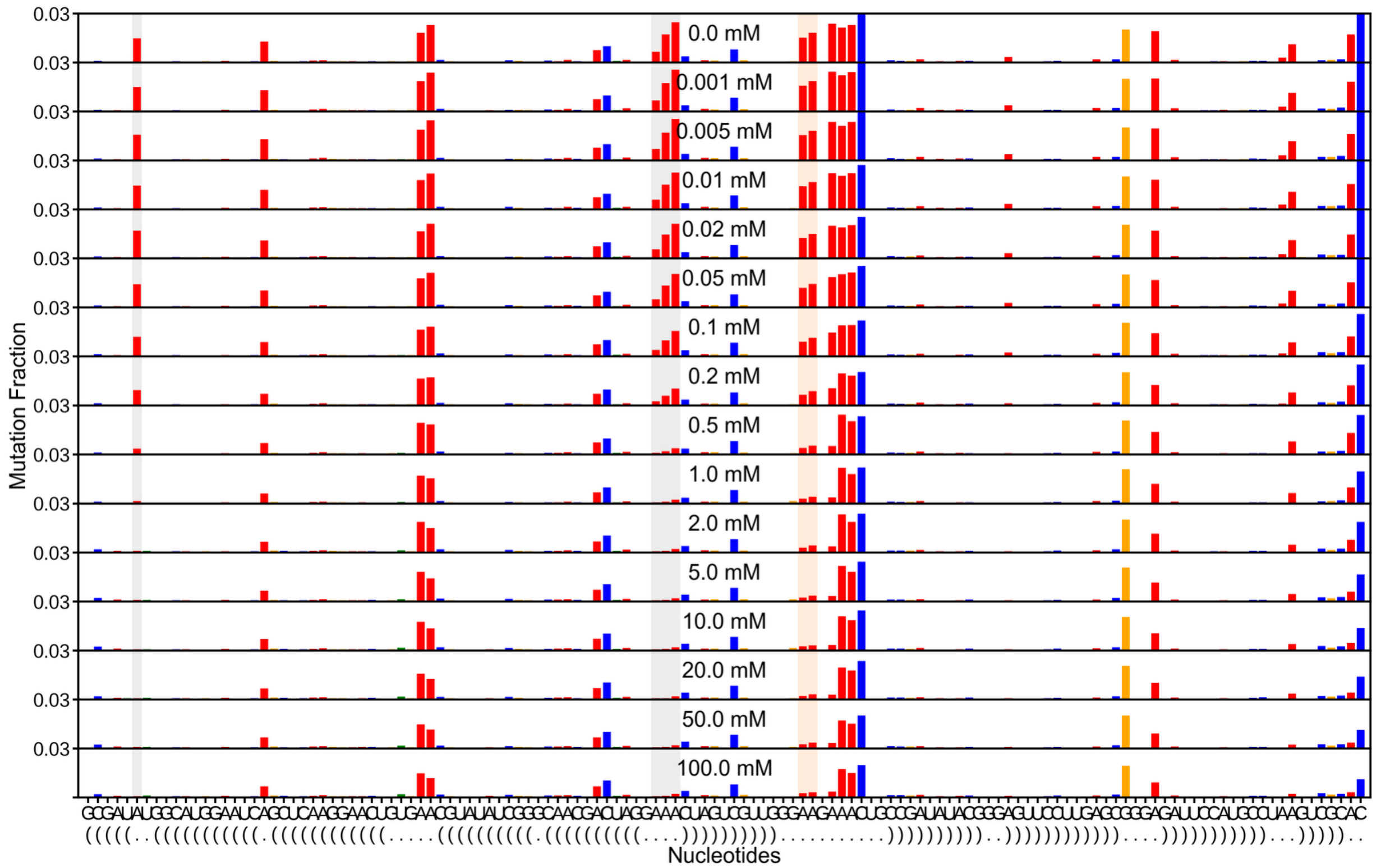

Figure S34: Wild type Mg^{2+} titration without AMP, replicate 1

Stacked population-average per-nucleotide reactivity at each Mg^{2+} concentration (0 to 100 mM) for Wild type without AMP; the y-axis is fixed to the construct's 99.5th-percentile reactivity. The TL/TLR reporters drop as Mg^{2+} drives docking. Replicate 1 of the Mg-titration whitelist.

bioRxiv preprint doi: https://doi.org/10.64898/2026.08.09.743768; this version posted August 11, 2026. The copyright holder for this preprint (which was not certified by peer review) is the author/funder, who has granted bioRxiv a license to display the preprint in perpetuity. It is made available under a CC-BY 4.0 International license.

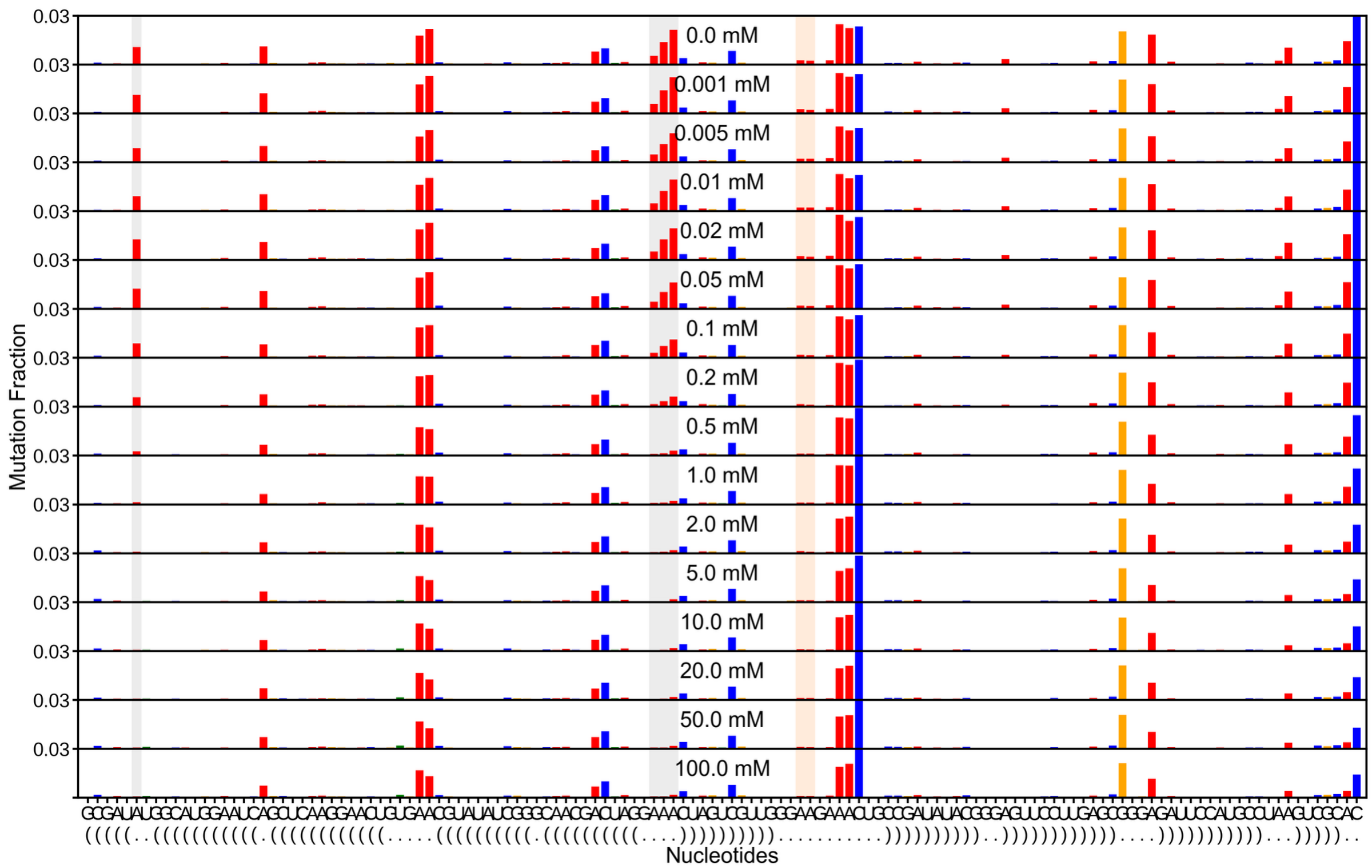

Figure S35: Wild type Mg^{2+} titration with saturating AMP, replicate 1

Stacked population-average per-nucleotide reactivity at each Mg^{2+} concentration (0 to 100 mM) for Wild type with saturating AMP; the y-axis is fixed to the construct's 99.5th-percentile reactivity. The TL/TLR reporters drop as Mg^{2+} drives docking. Replicate 1 of the Mg-titration whitelist.

bioRxiv preprint doi: https://doi.org/10.64898/2026.08.09.743768; this version posted August 11, 2026. The copyright holder for this preprint (which was not certified by peer review) is the author/funder, who has granted bioRxiv a license to display the preprint in perpetuity. It is made available under a CC-BY 4.0 International license.

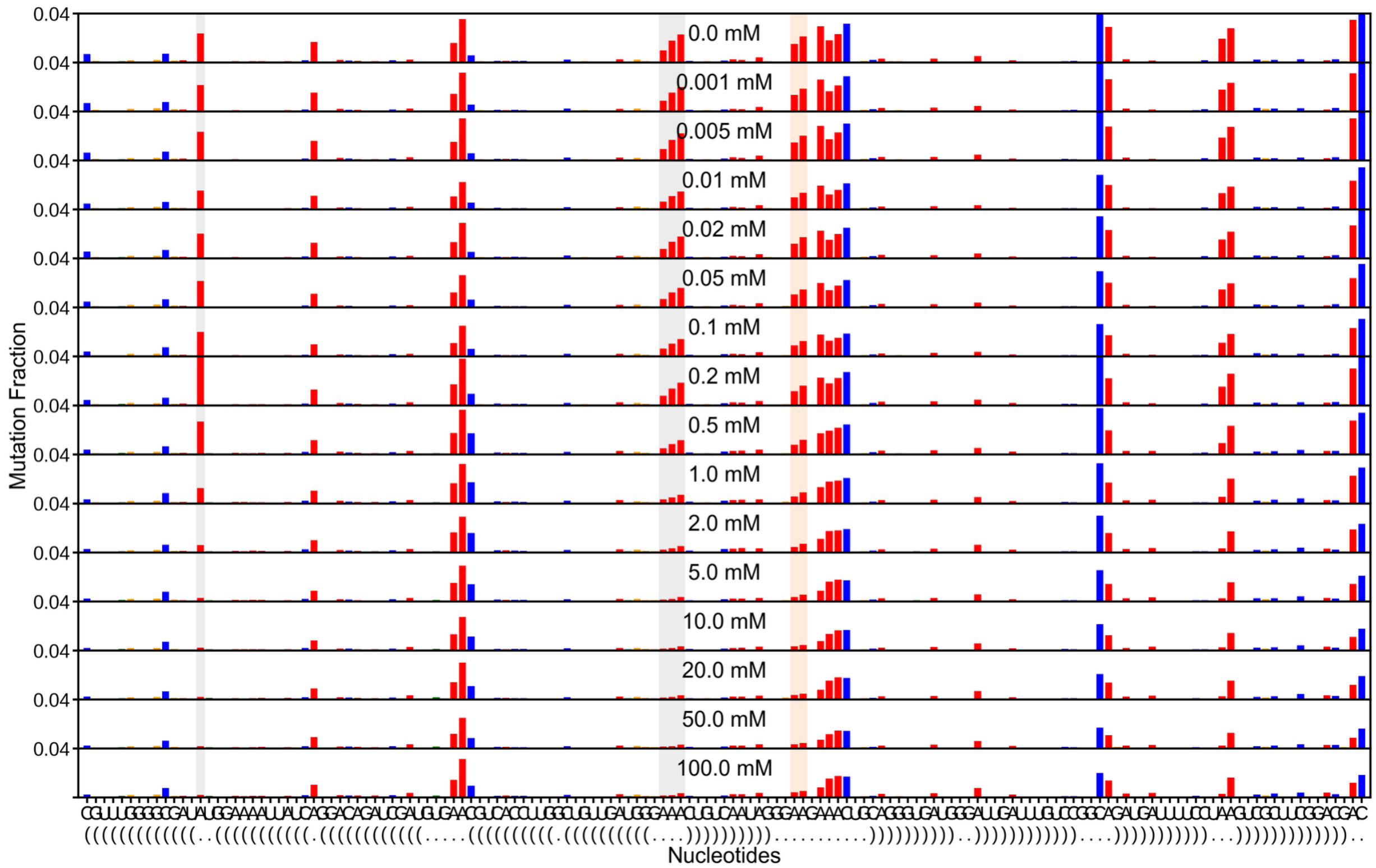

Figure S36: Always-On Mg^{2+} titration without AMP, replicate 1

Stacked population-average per-nucleotide reactivity at each Mg^{2+} concentration (0 to 100 mM) for Always-On without AMP; the y-axis is fixed to the construct's 99.5th-percentile reactivity. The TL/TLR reporters drop as Mg^{2+} drives docking. Replicate 1 of the Mg-titration whitelist.

bioRxiv preprint doi: https://doi.org/10.64898/2026.08.09.743768; this version posted August 11, 2026. The copyright holder for this preprint (which was not certified by peer review) is the author/funder, who has granted bioRxiv a license to display the preprint in perpetuity. It is made available under a CC-BY 4.0 International license.

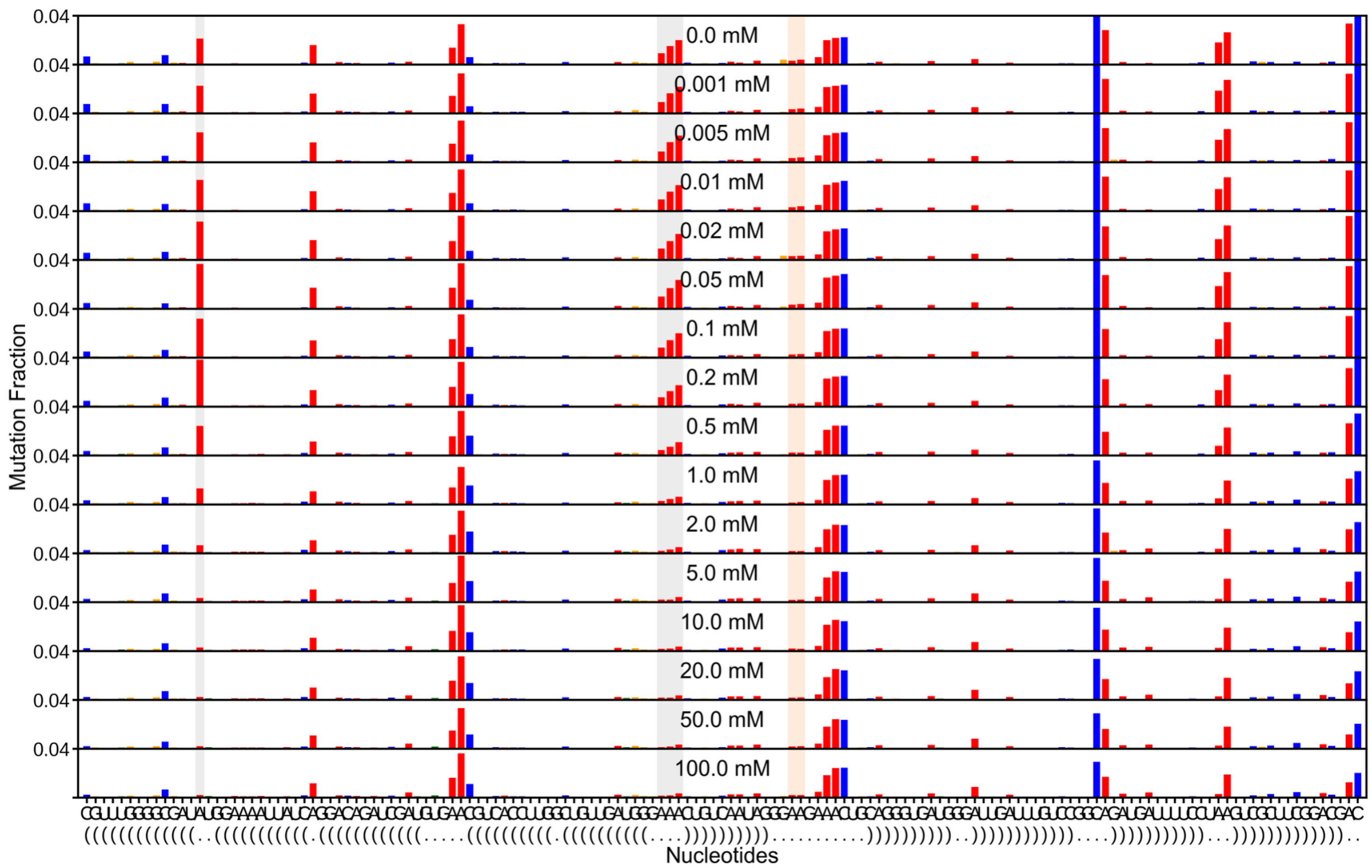

Figure S37: Always-On Mg^{2+} titration with saturating AMP, replicate 1

Stacked population-average per-nucleotide reactivity at each Mg^{2+} concentration (0 to 100 mM) for Always-On with saturating AMP; the y-axis is fixed to the construct's 99.5th-percentile reactivity. The TL/TLR reporters drop as Mg^{2+} drives docking. Replicate 1 of the Mg-titration whitelist.

bioRxiv preprint doi: https://doi.org/10.64898/2026.08.09.743768; this version posted August 11, 2026. The copyright holder for this preprint (which was not certified by peer review) is the author/funder, who has granted bioRxiv a license to display the preprint in perpetuity. It is made available under a CC-BY 4.0 International license.

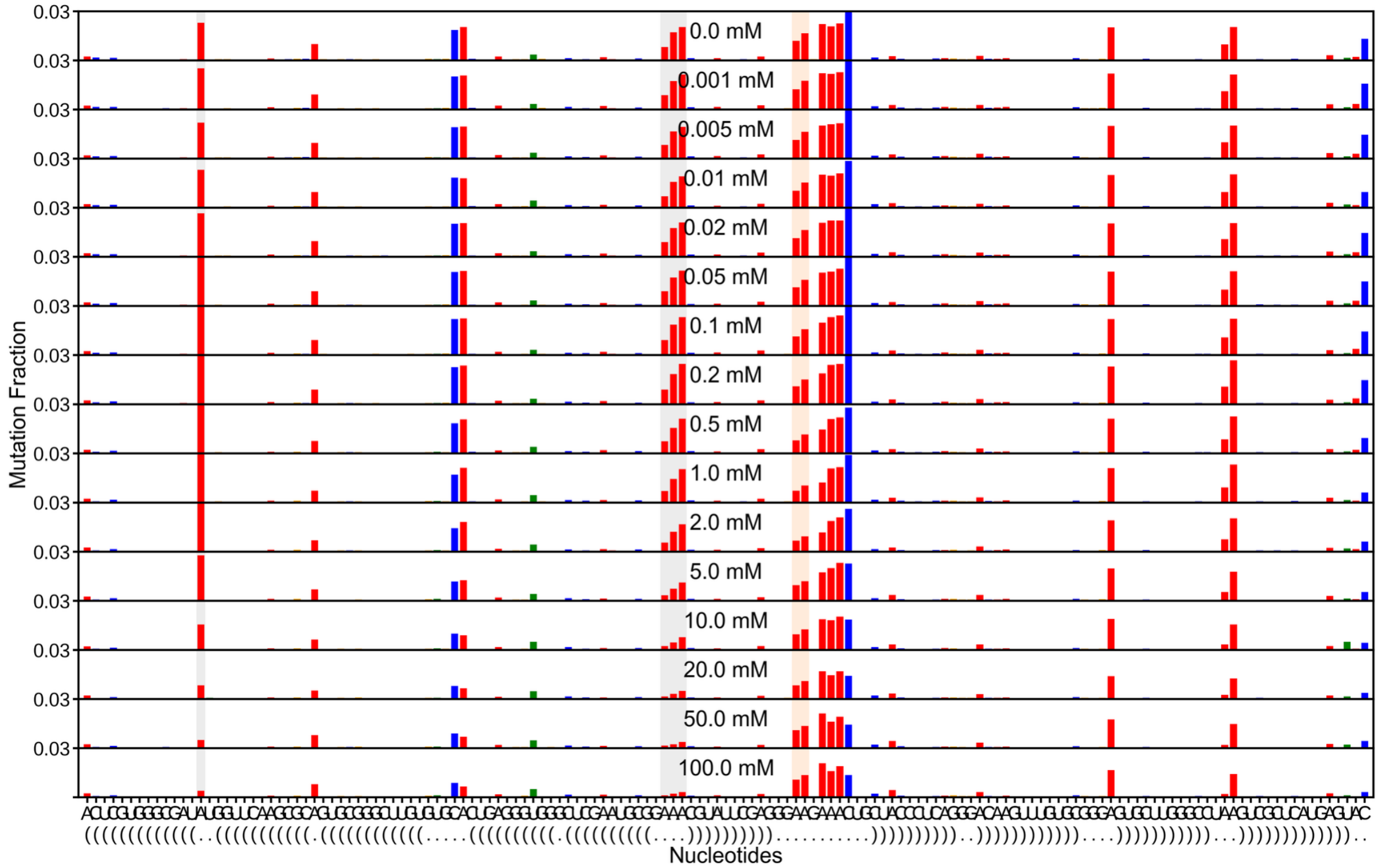

Figure S38: Always-Off Mg^{2+} titration without AMP, replicate 1

Stacked population-average per-nucleotide reactivity at each Mg^{2+} concentration (0 to 100 mM) for Always-Off without AMP; the y-axis is fixed to the construct's 99.5th-percentile reactivity. The TL/TLR reporters drop as Mg^{2+} drives docking. Replicate 1 of the Mg-titration whitelist.

bioRxiv preprint doi: https://doi.org/10.64898/2026.08.09.743768; this version posted August 11, 2026. The copyright holder for this preprint (which was not certified by peer review) is the author/funder, who has granted bioRxiv a license to display the preprint in perpetuity. It is made available under a CC-BY 4.0 International license.

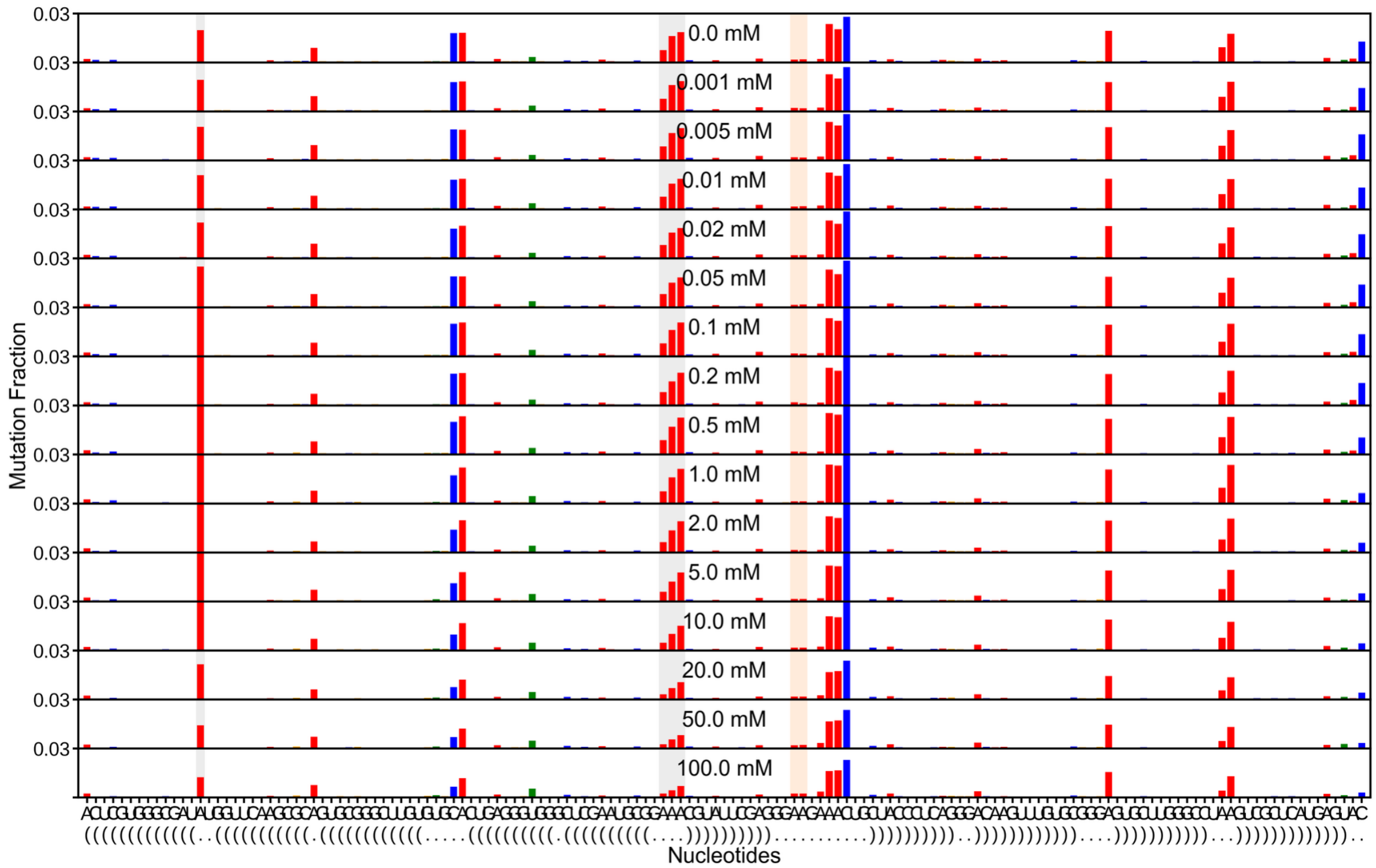

Figure S39: Always-Off Mg^{2+} titration with saturating AMP, replicate 1

Stacked population-average per-nucleotide reactivity at each Mg^{2+} concentration (0 to 100 mM) for Always-Off with saturating AMP; the y-axis is fixed to the construct's 99.5th-percentile reactivity. The TL/TLR reporters drop as Mg^{2+} drives docking. Replicate 1 of the Mg-titration whitelist.

bioRxiv preprint doi: https://doi.org/10.64898/2026.08.09.743768; this version posted August 11, 2026. The copyright holder for this preprint (which was not certified by peer review) is the author/funder, who has granted bioRxiv a license to display the preprint in perpetuity. It is made available under a CC-BY 4.0 International license.

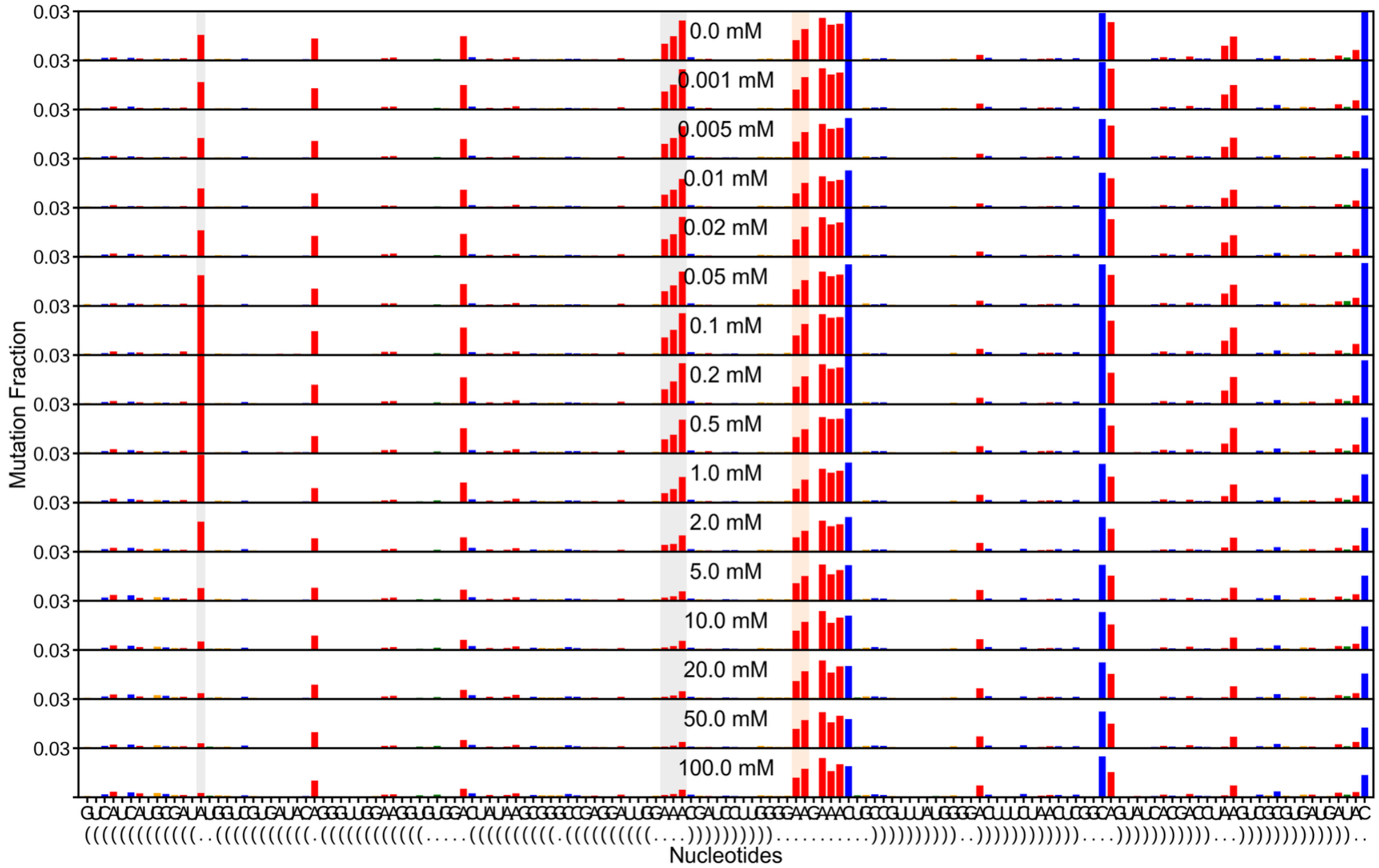

Figure S40: Switch-Off Mg^{2+} titration without AMP, replicate 1

Stacked population-average per-nucleotide reactivity at each Mg^{2+} concentration (0 to 100 mM) for Switch-Off without AMP; the y-axis is fixed to the construct's 99.5th-percentile reactivity. The TL/TLR reporters drop as Mg^{2+} drives docking. Replicate 1 of the Mg-titration whitelist.

bioRxiv preprint doi: https://doi.org/10.64898/2026.08.09.743768; this version posted August 11, 2026. The copyright holder for this preprint (which was not certified by peer review) is the author/funder, who has granted bioRxiv a license to display the preprint in perpetuity. It is made available under a CC-BY 4.0 International license.

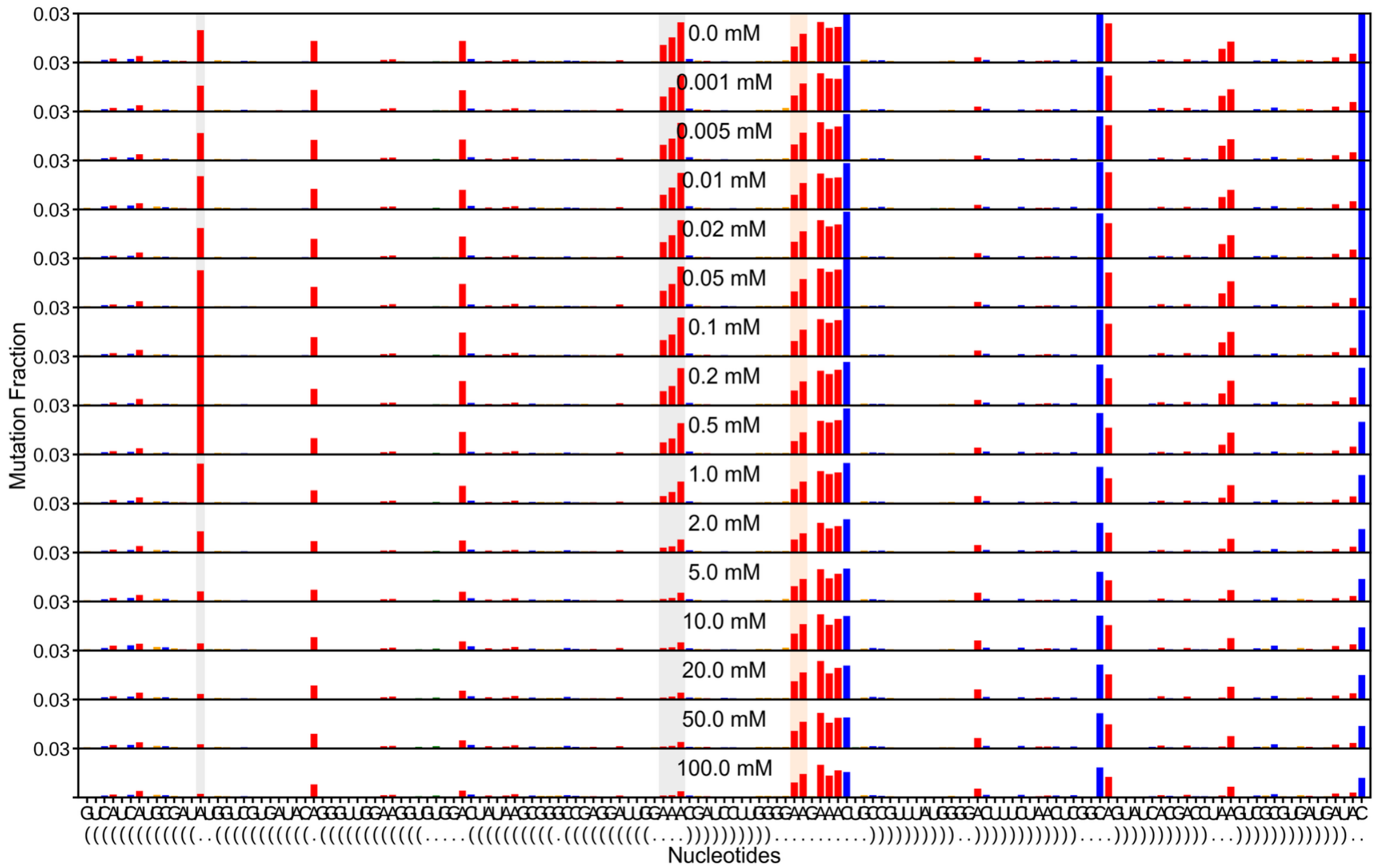

Figure S41: Switch-Off Mg^{2+} titration without AMP, replicate 2

Stacked population-average per-nucleotide reactivity at each Mg^{2+} concentration (0 to 100 mM) for Switch-Off without AMP; the y-axis is fixed to the construct's 99.5th-percentile reactivity. The TL/TLR reporters drop as Mg^{2+} drives docking. Replicate 2 of the Mg-titration whitelist.

bioRxiv preprint doi: https://doi.org/10.64898/2026.08.09.743768; this version posted August 11, 2026. The copyright holder for this preprint (which was not certified by peer review) is the author/funder, who has granted bioRxiv a license to display the preprint in perpetuity. It is made available under a CC-BY 4.0 International license.

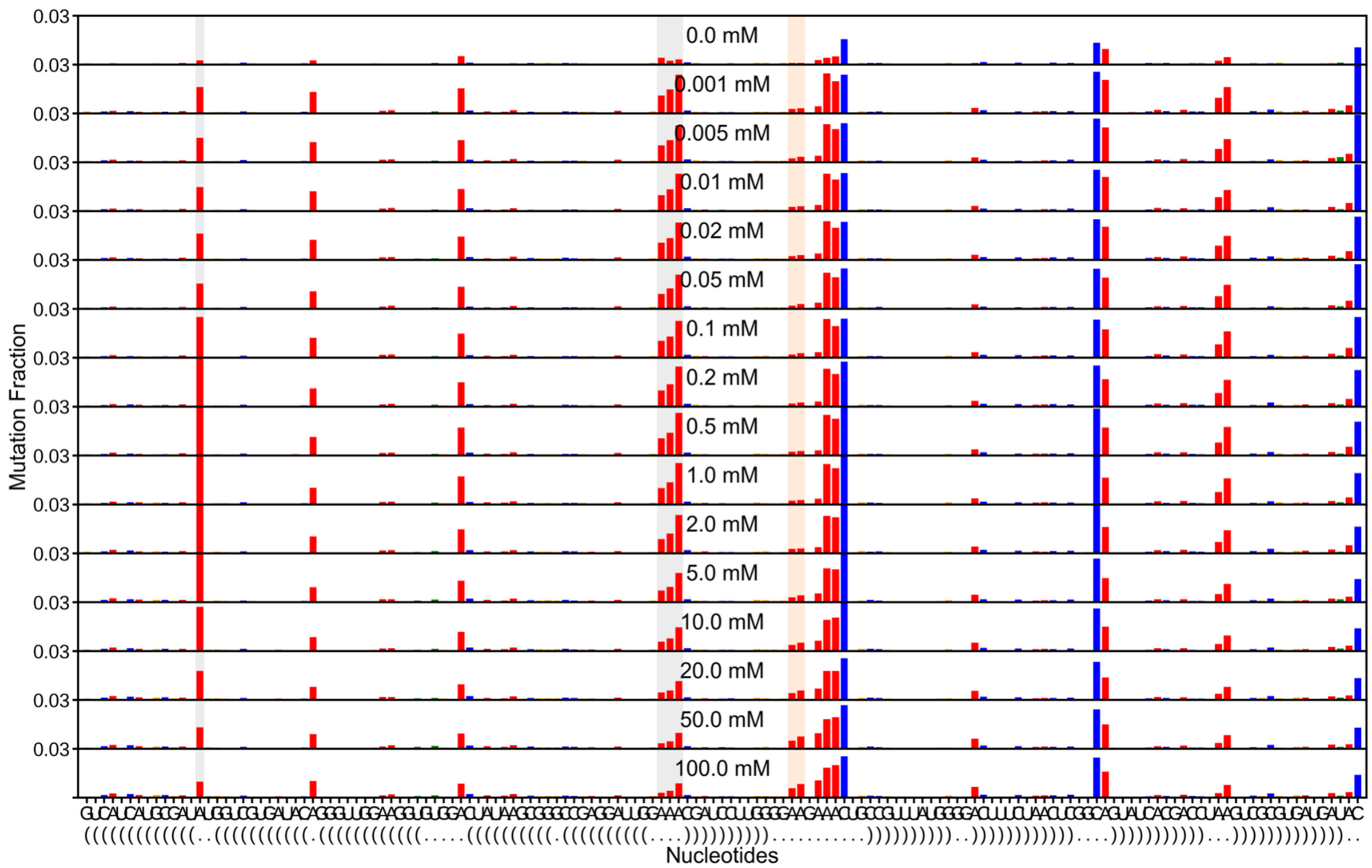

Figure S42: Switch-Off Mg^{2+} titration with saturating AMP, replicate 1

Stacked population-average per-nucleotide reactivity at each Mg^{2+} concentration (0 to 100 mM) for Switch-Off with saturating AMP; the y-axis is fixed to the construct's 99.5th-percentile reactivity. The TL/TLR reporters drop as Mg^{2+} drives docking. Replicate 1 of the Mg-titration whitelist.

bioRxiv preprint doi: https://doi.org/10.64898/2026.08.09.743768; this version posted August 11, 2026. The copyright holder for this preprint (which was not certified by peer review) is the author/funder, who has granted bioRxiv a license to display the preprint in perpetuity. It is made available under a CC-BY 4.0 International license.

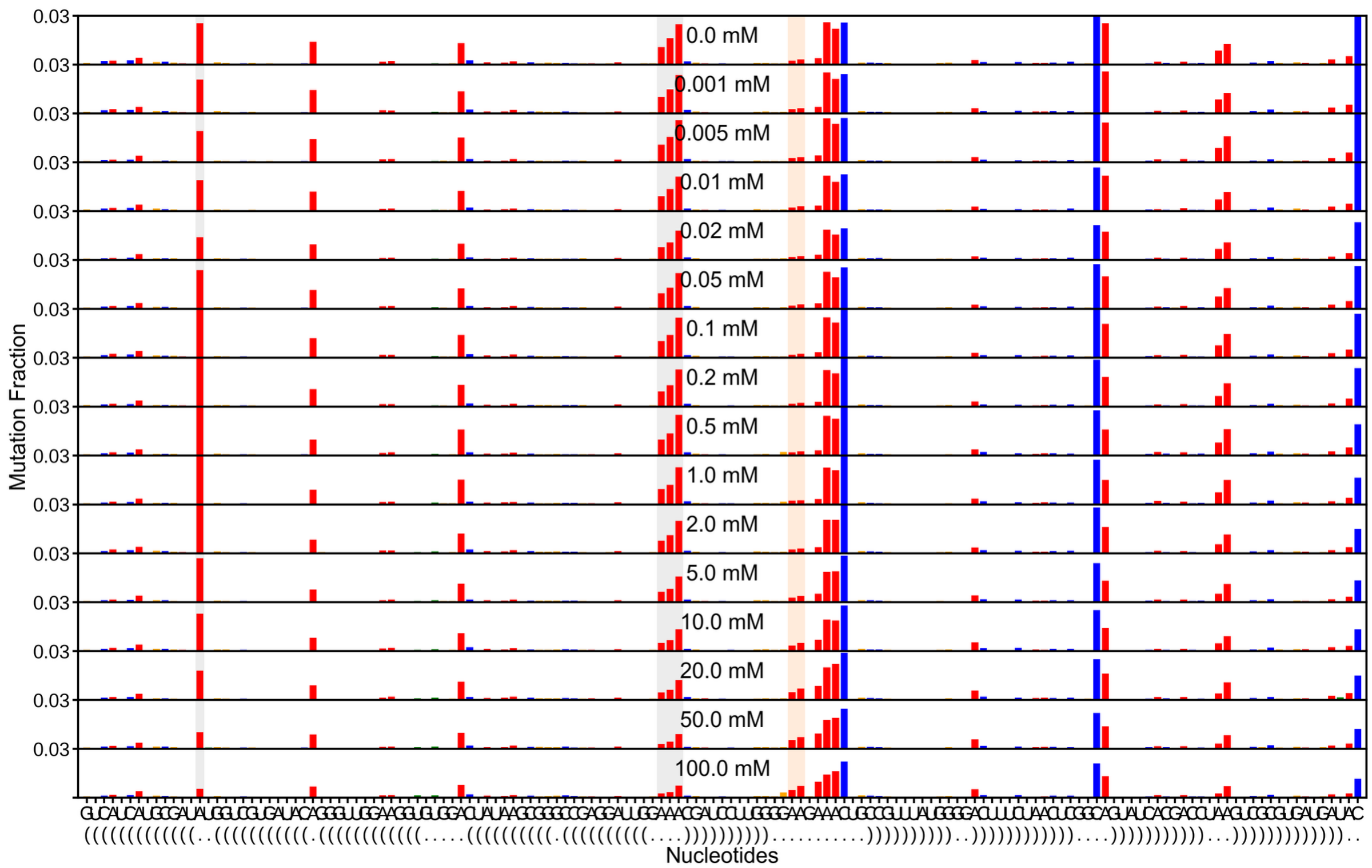

Figure S43: Switch-Off Mg^{2+} titration with saturating AMP, replicate 2

Stacked population-average per-nucleotide reactivity at each Mg^{2+} concentration (0 to 100 mM) for Switch-Off with saturating AMP; the y-axis is fixed to the construct's 99.5th-percentile reactivity. The TL/TLR reporters drop as Mg^{2+} drives docking. Replicate 2 of the Mg-titration whitelist.

bioRxiv preprint doi: https://doi.org/10.64898/2026.08.09.743768; this version posted August 11, 2026. The copyright holder for this preprint (which was not certified by peer review) is the author/funder, who has granted bioRxiv a license to display the preprint in perpetuity. It is made available under a CC-BY 4.0 International license.

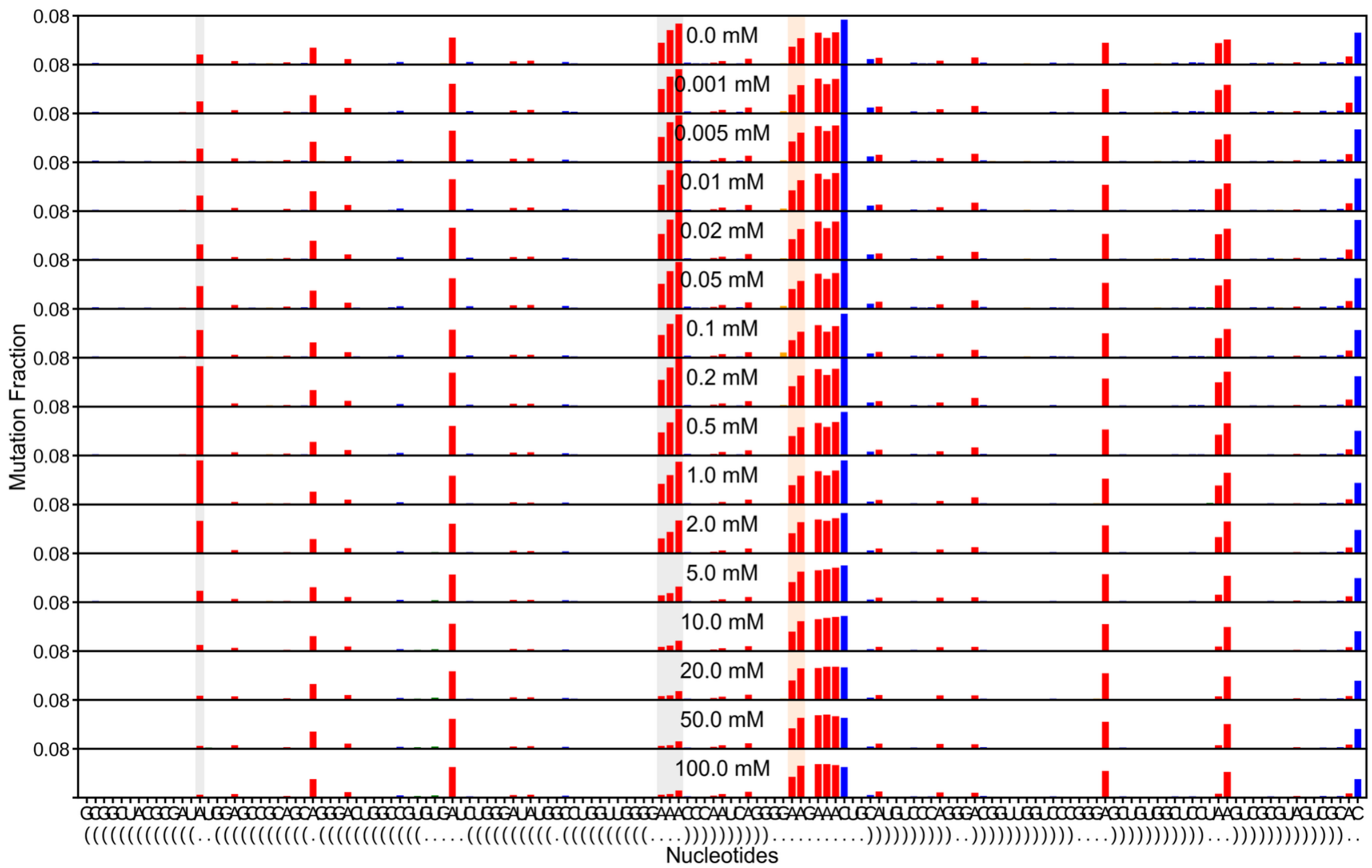

Figure S44: Switch-On Mg^{2+} titration without AMP, replicate 1

Stacked population-average per-nucleotide reactivity at each Mg^{2+} concentration (0 to 100 mM) for Switch-On without AMP; the y-axis is fixed to the construct's 99.5th-percentile reactivity. The TL/TLR reporters drop as Mg^{2+} drives docking. Replicate 1 of the Mg-titration whitelist.

bioRxiv preprint doi: https://doi.org/10.64898/2026.08.09.743768; this version posted August 11, 2026. The copyright holder for this preprint (which was not certified by peer review) is the author/funder, who has granted bioRxiv a license to display the preprint in perpetuity. It is made available under a CC-BY 4.0 International license.

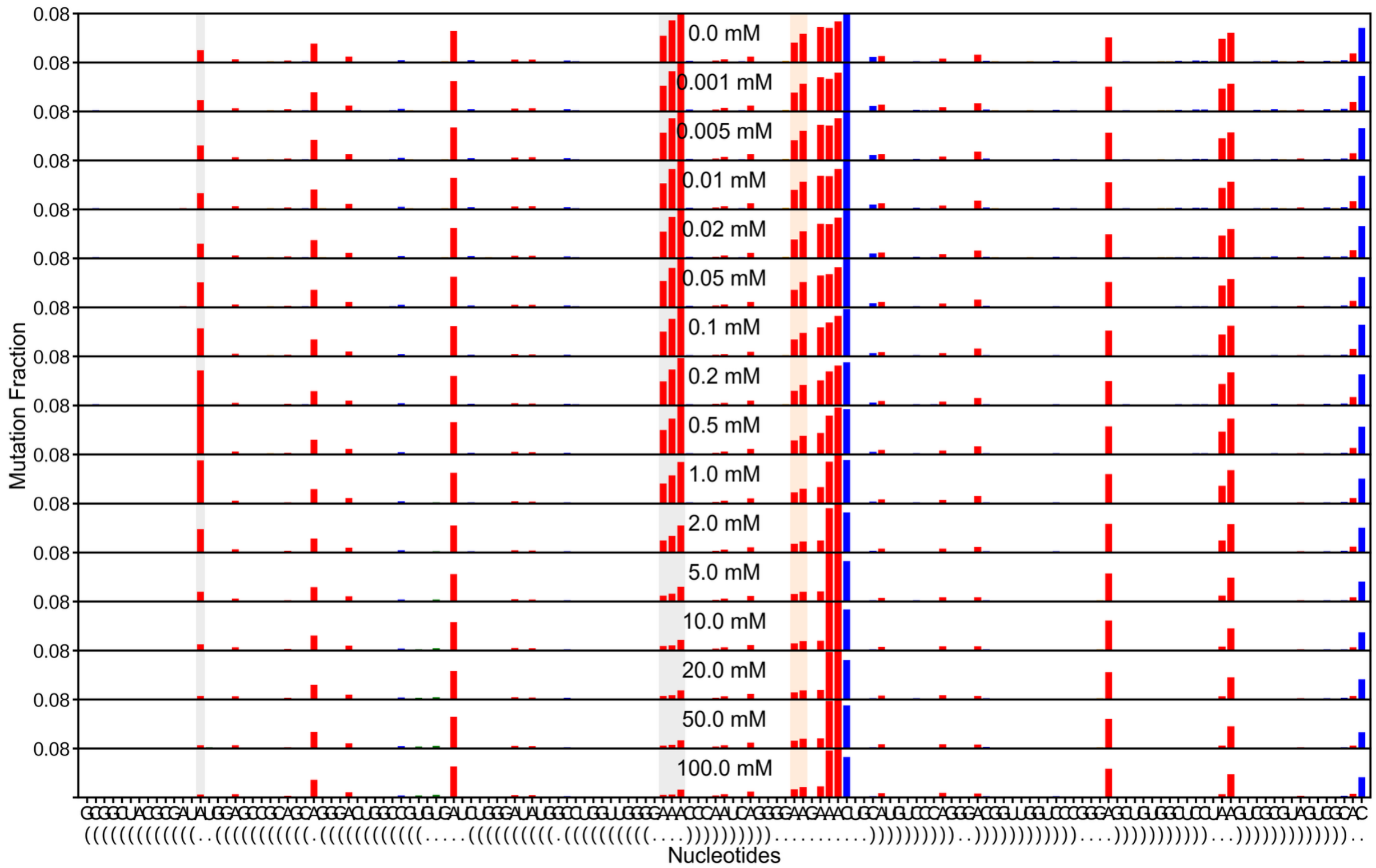

Figure S45: Switch-On Mg^{2+} titration with saturating AMP, replicate 1

Stacked population-average per-nucleotide reactivity at each Mg^{2+} concentration (0 to 100 mM) for Switch-On with saturating AMP; the y-axis is fixed to the construct's 99.5th-percentile reactivity. The TL/TLR reporters drop as Mg^{2+} drives docking. Replicate 1 of the Mg-titration whitelist.

bioRxiv preprint doi: https://doi.org/10.64898/2026.08.09.743768; this version posted August 11, 2026. The copyright holder for this preprint (which was not certified by peer review) is the author/funder, who has granted bioRxiv a license to display the preprint in perpetuity. It is made available under a CC-BY 4.0 International license.

Supplemental Tables

Table S1: Construct sequences

Sequences of the wild-type nanostructure and every library and representative construct, with their targeted mutations. Provided as the external spreadsheet Sequences.xlsx.

bioRxiv preprint doi: https://doi.org/10.64898/2026.08.09.743768; this version posted August 11, 2026. The copyright holder for this preprint (which was not certified by peer review) is the author/funder, who has granted bioRxiv a license to display the preprint in perpetuity. It is made available under a CC-BY 4.0 International license.

Table S2: Primer sequences

All DNA primers used for oligo-pool amplification, primer-assembly template construction, and reverse-transcription barcoding. Provided as the external spreadsheet Primers.xlsx.
